# Integrated proteomic, transcriptomic, and metabolomic profiling reveals that the gibberellin–abscisic acid hub runs flower development in the Chinese orchid *Cymbidium sinense*

**DOI:** 10.1093/hr/uhae073

**Published:** 2024-03-12

**Authors:** Sagheer Ahmad, Chuqiao Lu, Jie Gao, Yonglu Wei, Qi Xie, Jianpeng Jin, Genfa Zhu, Fengxi Yang

**Affiliations:** Guangdong Key Laboratory of Ornamental Plant Germplasm Innovation and Utilization, Environmental Horticulture Research Institute, Guangdong Academy of Agricultural Sciences, Guangzhou 510640, China; Guangdong Key Laboratory of Ornamental Plant Germplasm Innovation and Utilization, Environmental Horticulture Research Institute, Guangdong Academy of Agricultural Sciences, Guangzhou 510640, China; Guangdong Key Laboratory of Ornamental Plant Germplasm Innovation and Utilization, Environmental Horticulture Research Institute, Guangdong Academy of Agricultural Sciences, Guangzhou 510640, China; Guangdong Key Laboratory of Ornamental Plant Germplasm Innovation and Utilization, Environmental Horticulture Research Institute, Guangdong Academy of Agricultural Sciences, Guangzhou 510640, China; Guangdong Key Laboratory of Ornamental Plant Germplasm Innovation and Utilization, Environmental Horticulture Research Institute, Guangdong Academy of Agricultural Sciences, Guangzhou 510640, China; Guangdong Key Laboratory of Ornamental Plant Germplasm Innovation and Utilization, Environmental Horticulture Research Institute, Guangdong Academy of Agricultural Sciences, Guangzhou 510640, China; Guangdong Key Laboratory of Ornamental Plant Germplasm Innovation and Utilization, Environmental Horticulture Research Institute, Guangdong Academy of Agricultural Sciences, Guangzhou 510640, China; Guangdong Laboratory for Lingnan Modern Agriculture, Guangzhou 510640, China; Guangdong Key Laboratory of Ornamental Plant Germplasm Innovation and Utilization, Environmental Horticulture Research Institute, Guangdong Academy of Agricultural Sciences, Guangzhou 510640, China; Guangdong Laboratory for Lingnan Modern Agriculture, Guangzhou 510640, China

## Abstract

The seasonal flowering Chinese *Cymbidium* produce an axillary floral meristem and require a dormancy period during cold conditions for flower development. However, the bud activation mechanism remains elusive. This study evaluates the multi-omics across six stages of flower development, along with functional analysis of core genes to decipher the innate mechanism of floral bud initiation and outgrowth in the Chinese orchid *Cymbidium sinense*. Transcriptome and proteome analyses identified 10 modules with essential roles in floral bud dormancy and activation. Gene clusters in the early stages of flower development were mainly related to flowering time regulation and meristem determination, while the late stages were correlated with hormone signaling pathways. The metabolome identified 69 potential hormones in which gibberellin (GA) and abscisic acid (ABA) were the main regulatory hubs, and GA4 and GA53 exhibited a reciprocal loop. Extraneous GA application caused rapid elongation of flower buds and promoted the expression of flower development genes. Contrarily, exogenous ABA application extended the dormancy process and ABA inhibitors induced dormancy release. Moreover, *CsAPETALA1* (*CsAP1*) was identified as the potential target of ABA for floral bud activation. Transformation of *CsAP1* in *Arabidopsis* and its transient overexpression in *C. sinense* protoplasts not only affected flowering time and floral organ morphogenesis in *Arabidopsis* but also orchestrated the expression of flowering and hormone regulatory genes. The presence of ABA response elements in the *CsAP1* promoter, rapid downregulation of *CsAP1* after exogenous ABA application, and the activation of the floral bud after ABA inhibitor treatment suggest that ABA can control bud outgrowth through *CsAP1*.

## Introduction

Orchidaceae is one of the largest flowering plant families, distributed throughout the world [1, 2]. It is the largest family of monocots, comprising ~800 genera with >250 000 species [3]. In most orchids, the process of floral development, from initiation to flower opening, takes ~6–7 months. Flower bud differentiation in the widely recognized orchids *Phalaenopsis*, *Dendrobium*, and *Cymbidium* typically initiates between July and October. As the temperature drops during winter, the flower stalks gradually elongate and bloom between February and May of the following year [3]. While the degree of dependence on low temperature varies among different orchid varieties, cold temperature helps them escape bud abortion during semi-endodormancy.

Perennial plants use bud dormancy to survive the winter in temperate climates. This process is regulated by a series of hormonal, transcriptional, epigenetic, and physiological changes [4]. The progression of the dormancy cycle in plants is largely influenced by the mobility of molecules such as sugars, auxin, *Flowering Locus T* (*FT*) regulation, and possibly abscisic acid (ABA) [5]. Plasmodesmata permeability may be involved in dormancy control, because a short-day photoperiod can initiate ABA-mediated accumulation of callose [6]. Dormancy-associated MADS-box (DAM) transcription factors are the potential markers of dormancy in woody trees [7]. However, no DAM orthologs are found in the orchid genomes [3], suggesting a different plan in orchids than the conduits regulated by *SVP*/*StMADS11* in other perennial species, such as Rosaceae species, *Populus trichocarpa*, and kiwifruit [8, 9]. Why flower buds cannot grow continuously, needing a dormancy pause to continue growth and blooming, has always been an unsolved mystery in orchids. Therefore, comprehending the mechanisms that regulate the physiological events during flower bud dormancy and control blooming time is crucial.

Flower development is a complex process regulated by multiple intrinsic and extrinsic agents. Plant hormones play indispensable roles in floral transition and continuous development. Gibberellin (GA) significantly affects the flowering process [10]. There are more than130 known GAs, but only a few are biologically active, including GA1 and GA3–GA7 [11]. Biosynthesis of GA begins with plastid-localized GGDP (geranylgeranyl diphosphate), which changes into *ent*-kaurene [12]. In the endoplasmic reticulum, *ent*-kaurene is oxidized by cytochrome P450 mono-oxygenase to produce GA12 [13]. From this point, two parallel pathways run in the cytoplasm, including 13-hydroxylation and non-13-hydroxylation. Three groups of 2-oxoglutarate-dependent dioxygenases regulate the catalytic activity [12]. Among these, GA20oxs (GA20-oxidases) produce GA precursors, GA3oxs (GA3-oxidases) produce bioactive GAs, and GA2oxs (GA2-oxidases) irrevocably disable precursors and bioactive GAs by 2β-hydroxylation [14]. The dominance of either of the pathways depends upon species, stage of development, and organ type [11]. For example, GA1 is dominant during the non-reproductive growth of rice, but GA4 dominates during anthesis [15, 16]. In *Arabidopsis*, GA4 regulates flowering [17]. Maintaining a low concentration of GA4 is crucial to stop axillary bud activation and outgrowth [11].

GA and ABA act antagonistically during plant developmental processes. Release of bud dormancy is positively correlated with the reduction of ABA levels [18]. Exogenous ABA application causes flowering time fluctuations, indicating that ABA might function as an internal element influencing floral transition [19]. The ABA-deficient mutants (*aba1* and *aba2-4*) show late flowering in *Arabidopsis* [20]. However, ABA also plays inhibitory roles. For example, ABI4 (ABA-INSENSITIVE 4), a key ABA signaling pathway component, negatively controls floral transition through *FLC* activation in *Arabidopsis* [21]. These contradictory responses of ABA could be influenced by many variables, such as growth condition, photoperiod sensitivity, ABA concentration, and species-specific effectors involving the crosstalk of flowering pathways and ABA. ABA is also involved in floral bud regulation through key floral integrators, such as *AP1*. Exogenous ABA application upregulates *AP1* expression in litchi, whereas the ABA biosynthesis inhibitor downregulates it [22].

*APETALA1* (*AP1*) is a floral meristem identity gene that plays a crucial role to specify floral meristem during floral transition [23]. It targets flowering time genes encoding MADS-box transcription factors to regulate floral meristem identity and floral patterning [23, 24]. The molecular networks underlying the function of *AP1* in floral organ design and floral transition have been researched extensively. The *ap1* mutation in *Arabidopsis* causes the absence of petals, petal transformation into bract-like structures, and the production of axillary flowers [25]; however, its overexpression causes early flowering and transformation of shoot apical meristem into floral meristem [26]. So far, *AP1* orthologs have been identified in a number of species, such as apple [27], pea [27], longan [28], common wheat [29], birch [30], trifoliate orange [31], poplar [32], and moth orchid [33]. Conserved function of *AP1* orthologs in the flowering process and floral organ formation has been found in different plants. For example, a tree ortholog of *Arabidopsis AP1*, *LAP1* (*Like-AP1*), acts as a link between *FT* and *AIL1* (*AINTEGUMENTA-like 1*), and its downregulation is essential for short-day-induced growth cessation [34]. The ectopic expression of the *Fortunella crassifolia AP1* gene in *Arabidopsis* [35] and heterologous overexpression of *Betula pendula* and *Pisum sativum AP1*-like genes in *Nicotiana tabacum* cause early flowering. Overexpression of *Dendrobium* orchid *AP1* (*DOAP1*) results in early flowering and termination of inflorescence meristem into floral meristem [36]. However, more and more subfunctional differentiation or new mechanisms of action of paralogous genes have been found for *AP1* homologous genes [3]. It indicates that there are many species-specific unknown functions or mechanisms of *AP1* homologous genes to be discovered.

Chinese *Cymbidium* have a long history of cultivation in China and embody profound cultural connotations, making them particularly significant in the traditional Chinese flower market. *Cymbidium sinense* is a magnificent ornamental orchid that has a significant cultural history and aesthetic value in China. The plant features elegant flowering spikes with scented flowers and attractive dark green foliage, making it a popular choice among gardeners. Recently, the first high-quality genome has been assembled to the chromosome level for *C. sinense* and the unigenes related to flower development have been studied [3]. However, the specific pathways regulating early stages of flower development and the role of hormones have not been explained.

To comprehensively grasp the regulation of bud development progression, we integrated cytological, transcriptome, proteome, and metabolome data to obtain a holistic view of the key regulatory networks governing flower development. Initially, the comparative transcriptome and proteome analyses yielded a substantial dataset of essential floral and hormonal regulators and then subsequent experiments further validated the specific role of the GA–ABA loop in flower development regulation through its interaction with floral integrators.

## Results

### Flowering physiology and intermittent floral development of *Cymbidium sinense*

**Figure 1:**
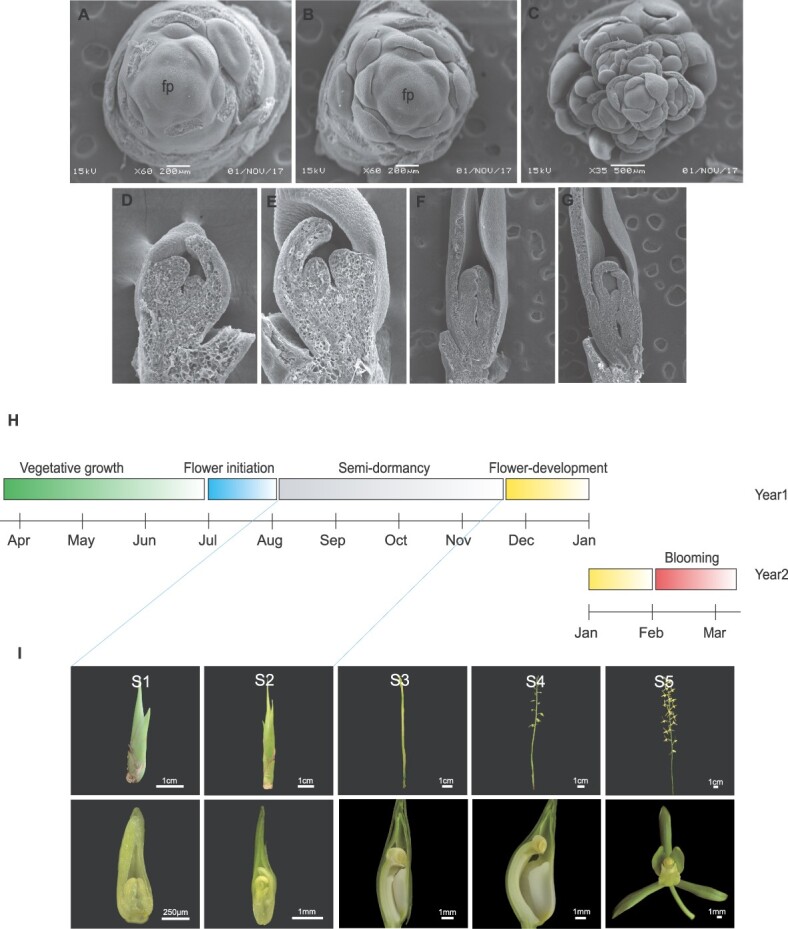
Complete process of flower development of *C. sinense* and seasonal distribution of different development stages. **A**, **B** First stage of flower development (S1). Flower development starts with the appearance of a floral buttress on the shoot flank that forms a flower primordium (fp). **C**–**E** Differentiation of the flower primordia into sepal, petal, lip, and column primordia. **F**, **G** Second stage of flower development (S2). The second stage denotes slow elongation of the inflorescence meristem for 3–4 months. **H** Complete cycle of flowering from vegetative to reproductive phase transition and blooming in the next year. The vegetative growth lasts >2 years and then transition occurs in July. After a short period of flower initiation, the floral buds enter a period of semidormancy that lasts >3 months. Flower development resumes in November and blooming occurs in February of the next year. **I** Visual display of five stages of flower development (S1–S5). S1 and S2 undergo semidormancy before resuming flower development from S3. S4 represents complete flower formation and S5 denotes fully opened flowers. Lower row shows the vertical dissection of floral bud from S1 to S4, while S5 shows the complete flower with its organs.

In July, flower development starts with the appearance of a floral buttress on the shoot flank that forms a flower primordium (Fig. 1A and B), which is stage 1. In this stage, the apical meristem grows rapidly and forms the inflorescence primordia within 2 weeks, which contain ~10 floret primordia. The flower primordia gradually differentiate into sepal, petal, lip, and column primordia (Fig. 1C–E).

The second stage lasts for 3–4 months. The inflorescence elongates very slowly and remains below 5 cm (Fig. 1F and G). The sepal elongates rapidly and forms thin strips, which are longer than other flower organs. The petals are comparable in length to the labellum and stick tightly to the column. The anther becomes bigger and mature, but the body of the column maintains slow growth speed, which lasts >4 months.

Flower development speeds up in S3, the flower stem elongates, and the inflorescence and flower emerge from the bract. The length of the petal and lip exceeds the column at this stage. The column further elongates and bends, and the anthers appear yellow. During S4, in mid-January, the floral organs grow more and mature, the lobes spread, and the column keeps bending. The entire flower grows and blooms after a week (S5).

This process takes ~4 months and then flower development takes about one and a half months before blooming in the following year (Fig. 1H and I).

The flower bud enters the slow growth phase (lasting >3 months). Compared with continuous flowering cultivars, the development of the lip and column of seasonally flowering *C. sinense* remains suspended. Moreover, the labellum does not develop during the dormancy stages, indicating that the genes controlling the development of petals and labellum are different, and the differentiation and development of flower organs are not synchronized.

### Two-omics data and functional enrichments

**Figure 2:**
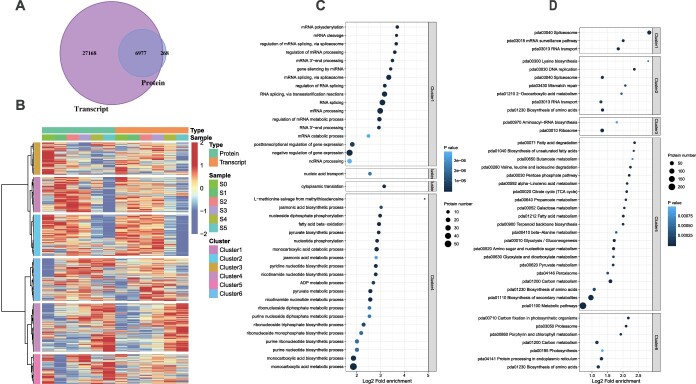
Overview of proteome and transcriptome data with annotations. **A** Comparison of proteome and transcriptome data to find transcripts with corresponding protein concentrations. The Venn diagram shows that a total of 6977 genes were identified both at transcriptome and proteome levels. **B** Cluster analysis of proteins and transcripts. The hclust ‘ward.D’ method was used to perform cluster analysis on the expression levels of genes in two dimensions. The cluster analysis divided proteins or transcripts into six categories/clusters. **C**, **D** GO (**C**) and KEGG (**D**) annotation analyses of six clusters. Fisher’s exact test was used to analyze the GO and KEGG enrichment of these categories and the top 40 significantly enriched (*P* < 0.05) functional categories are shown in the figures.

To reveal the regulatory network of this unique pattern of intermittent floral organ development of *C. sinense*, we performed transcriptome and proteome analyses and identified 35 551 transcripts and 7245 proteins, respectively. By comparing the transcriptome and proteome data, we identified 6977 genes that were present at both transcriptome and proteome levels (Fig. 2A). High Pearson’s correlation coefficients (*R*^2^ > 0.9) among the three biological replicates (Supplementary Data Fig. S1) show the quality control. Principal component analysis (PCA) shows repeatability between samples (Supplementary Data Fig. S2). A high level of similarity among replicates shows that the experiment was conducted under good control (Supplementary Data 1).

The potential relationships between genes at the transcriptional and translational levels were explored for different developmental stages (0–5) (Fig. 2B). First, through the one-to-one correspondence between transcripts and proteins, the proteome and transcriptome expression datasets were merged. Then the hclust ‘ward.D’ method was used to perform cluster analysis on the expression levels of genes in two dimensions. These proteins or transcripts were divided into six categories. In the first three clusters (1–3), the genes were mainly upregulated in the early stages of flower development and downregulated in the late stages of flower development (Fig. 2B). Contrarily, the genes in clusters 4–6 showed a general downregulation trend in the early stages of flower development and an upregulation trend in the late stages of flower development. Moreover, the gene expression trends were mostly correlated with the protein concentrations.

We used Fisher’s exact test to analyze the GO (Supplementary Data Table S1) and KEGG (Supplementary Data Table S2) enrichment of these categories and the top 40 significantly enriched (*P* < 0.05) functional categories were selected (Fig. 2C and D). The highly enriched biological process categories with high log_2_ fold enrichment included RNA splicing, mRNA processing, monocarboxylic acid catabolic process, monocarboxylic acid metabolic process, monocarboxylic acid biosynthesis process, and cytoplasmic translation (Fig. 2C). The KEGG pathways with a significant number of gene enrichments, high log_2_ fold enrichment, and low *P*-value included spliceosome (pda03040), biosynthesis of secondary metabolites (pda01110), carbon metabolism (pda01200), and ribosome (pda03040) (Fig. 2D).

### Quantitative comparative analysis of protein and mRNA

**Figure 3:**
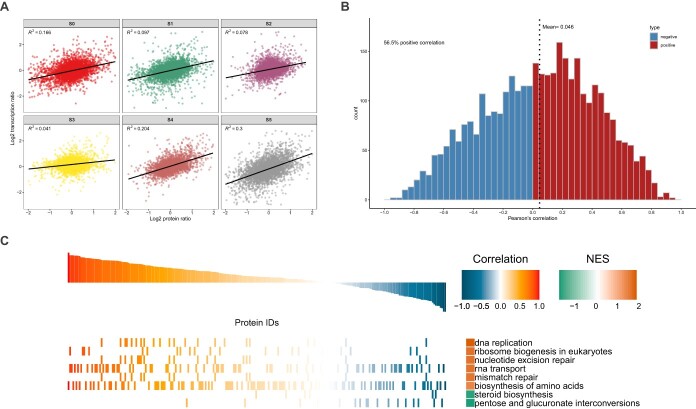
Quantitative comparative analysis of proteome and transcriptome data. **A** Scatter plots between transcripts and their corresponding protein concentrations. According to the corresponding relationship between protein and transcript IDs above, the protein expression level and mRNA expression level are combined. To unify the definition of expression, before drawing the scatter plot, the expression of both protein and transcript were log_2_-transformed. The horizontal axis in the figure is the protein expression level and the vertical axis is the transcript expression level. **B** Cumulative distribution of quantitative Pearson correlation coefficients between transcriptome and proteome. The abscissa represents the Pearson correlation coefficient and the ordinate represents the number of proteins or transcripts under the Pearson correlation coefficient. Pearson correlation coefficients >0 (positive correlation between protein and transcript) are indicated in red and Pearson correlation coefficients <0 (negative correlation between protein and transcript) are indicated in blue. **C** KEGG pathway analysis and GSEA based on quantitative correlation coefficients between transcriptome and proteome. KEGG annotations are used as gene sets with known functions, and the correlation coefficient between input proteins and transcripts is used for GSEA to study the main KEGG pathways involved in different regulatory relationships. Pathways with NOM *P* < 0.05 were screened to draw GSEA graphs. NES, corrected normalized enrichment score.

Comparison of the quantitative correlations of the two omics provides a quick insight into the underlying regulatory relationship between proteins and transcripts. Figure 3A shows the scatter plots between the transcripts and their corresponding protein expression levels. We further used the Pearson correlation coefficient to evaluate the relationship between each pair of transcripts and protein expression (Fig. 3B). Of the correlation coefficients between transcripts and protein expression, 56.5% are >0 and the average is 0.046. In addition to these positive regulatory relationships between the transcriptome and the proteome, there are 43.5% negative regulatory relationships between proteins and transcripts. Gene set enrichment analysis (GSEA) was performed using the KEGG annotations to reveal the pathways involved in proteins or transcripts under different regulatory relationships (Fig. 3C). Pathways with NOM *P* value <0.05 were screened for GSEA. DNA replication showed the highest positive correlation coefficient (FDR *q* value 0.05) and normalized enrichment score (NES) (1.93). Contrarily, steroid biosynthesis and pentose and glucuronate interconversions showed negative correlation and NES relationships (Supplementary Data Table S3).

### Stage-specific genes and proteins

Up- and downregulated transcripts and proteins were compared for flower developmental stages (Supplementary Data Fig. S3A and B). An increasing upregulation trend can be seen from floral development stage 0 (FD0) to FD3, and the highest numbers of upregulated transcripts were seen in FD3 compared with other stages, while the highest number of downregulated transcripts was observed in FD5 (Supplementary Data Fig. S3A). However, the proteome data showed highly upregulated proteins at FD2, while the early stages (FD0 and FD1) showed more downregulated proteins (Supplementary Data Fig. S3B). There was no certain trend in the proteome data compared with the transcriptome data. This shows an obvious difference at the level of gene expression and protein concentrations.

The top 10 highly expressed and correlated transcripts and proteins were identified to analyze the individual developmental stage. Among them, we found some important regulators for flower development (Supplementary Data Fig. S3C). Two lipoxygenase (LOX) transcripts and proteins were particularly expressed in FD0. *LOX* genes regulate the transition to flowering in *Arabidopsis* [37], suggesting a role for vegetative to reproductive transition. The other highly expressed proteins and transcripts were mainly related to cell structures and cytoskeleton. High levels of Hd3a transcript and protein were observed only in FD1. Hd3a is a key flowering time activator in rice. In the absence of Hd3a expression, the transcription of *AP1* orthologs, *MADS14* and *MADS14*, is strongly reduced [38], suggesting an important role of Hd3a in early flower development. Significantly high expression of histone deacetylase 2 (HDT2) was observed in FD2. HDT2 is mainly expressed in actively dividing tissues, such as apical meristems [39]. It controls cell division to expansion by fine-tuning GA metabolism [40]. The highly expressed genes in FD3 were mainly concerned with the energy-producing system, such as ATP synthase, RCA (Rubisco Activase), PSBS (Photosystem II Subunit S), and CAB36 (Chlorophyll a-b binding protein 36), and flavonoid pathway enzymes, such as CHS1 (Chalcone Synthase 1), FLS (Flavonol Synthase), and PAL (Phenylalanine Ammonia Lyase). This coincides with the rapid growth of FD3 and anthocyanin synthesis in this stage. During the development and opening of a flower, it accumulates many flavonoids, sugars, and other metabolites, as well as aroma components. Thus, in FD4 and FD5 the genes were mainly related to fatty acid metabolism (Supplementary Data Fig. S3C). It is worth noting that a strigolactone biosynthesis gene, CCD7 (Carotenoid cleavage dioxygenase 7), was specifically expressed in FD5. CCD genes can catalyze carotenoids to form aromatic volatile substances, which was positively correlated with the release of flower fragrance in FD5. These stage-specific genes correlated well with the distinct developmental characteristics of each stage.

### Weighted gene coexpression network analysis and core regulators of *Cymbidium sinense* flower development

**Figure 4:**
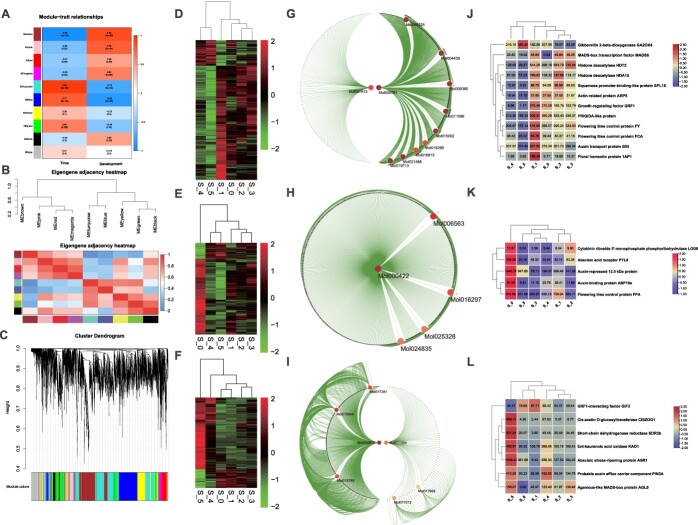
Weighted gene coexpression network analysis and identification of hub genes. **A** WGCNA modules for flowering time and flower development. A total of 6905 transcripts were run in the WGCNA and after the removal of bad genes with poor expression and low connectivity 5553 genes were selected for module construction. Cell colors show correlation coefficients; blue shows negative correlation and red shows positive correlation. *P*-values are shown in brackets. **B** Eigengene adjacency clustering of modules and eigengene adjacency heat map. **C** Clustering dendrograms of DEGs. Dissimilarity is based on topological overlap together with the assigned module colors. The tree leaves show the DEGs and the height indicates the closeness of individual genes. **D** Heat map showing the gene expression distribution of MEturquoise. **E** Heat map showing the gene expression distribution of MEblue. **F** Heat map showing the gene expression distribution of MEbrown. **G**–**I** Identification of hub genes using the cytoHubba application in Cytoscape for the modules MEturquoise (**G**), MEblue (**H**), and MEbrown (**I**). **J**–**L** Heat maps of the expression of hub genes with their annotations for the modules MEturquoise (**J**), MEblue (**K**), and MEbrown (**L**).

For weighted gene coexpression network analysis (WGCNA), transcripts were selected with corresponding protein concentrations. A total of 6905 transcripts were run in the WGCNA and after the removal of bad genes with poor expression and low connectivity 5553 genes were selected for module construction. A total of 10 modules were constructed for flowering time and flower development. Among these, MEturquoise contained highly correlated genes with a correlation coefficient of 0.93 (Fig. 4A), followed by MEblue (0.77). MEbrown (correlation coefficient −0.96) included the most downregulated genes for flowering time regulation, whereas it contained the most upregulated genes for flower development. The eigengene adjacency heat map also shows that MEturquoise and MEblue cluster together, while MEbrown is separate (Fig. 4B). The cluster dendrogram shows the specificity of different modules in the regulation of flowering time and flower development (Fig. 4C). The expression intensities of the genes from three key modules, MEturquoise, MEblue, and MEbrown, are shown as heat maps, showing stage specificity, in Fig. 4D–F. Specifically, the genes of MEturquoise showed significant expression in the early stages of flower development (Fig. 4D), especially FD1. For MEblue, the highest gene expression was observed in FD0 compared with other stages (Fig. 4E). However, the genes of MEbrown were mainly downregulated in the early stages (Fig. 4F) but showed high expression in the fast-growing FD4 and FD5, suggesting that flower development may work independently of flowering time regulation. From the three key modules, we isolated the essential genes that play important roles in flowering time regulation and flower development (Fig. 4G–I). For each module Cytoscape edges were produced, and the key genes were filtered. These hub genes showed extensive networking within the genomic data.

A total of 12 genes were selected from the MEturquoise module with upregulated expressions in the early stages and downregulation in the late stages (Fig. 4J). These genes were mainly related to hormonal and flowering time regulation, including floral time regulators FCA and FY, floral homeotic protein AP1, and auxin transport protein BIG, which were specifically expressed in FD1 compared with other stages. Similarly, five genes from MEblue also showed high expression in the early stages (Fig. 4K). These genes showed the highest expression in FD0 and contained flowering time regulator FPA and hormonal regulators for auxin, cytokini, and ABA. Seven genes from MEbrown showed higher expression in the late stages of flower development than in the early stages (Fig. 4L). Here, growth regulating factor (GRF1)-interacting factor along with auxin (PIN3A), cytokinin (CISZOG1), and gibberellin (KAO1) regulators were expressed specifically in FD5, followed by FD4 and FD3.

Through individual mining, we isolated 78 potential genes with their corresponding protein concentrations (Supplementary Data Fig. S4A). These are related to flower timing and development, and hormonal regulators, including ABA, GA, auxin, cytokinin, strigolactone, and ethylene. These genes have potential roles in floral regulatory pathways. WGCNA-based correlation analysis of these genes suggested that all the floral pathway regulators were connected with each other (Supplementary Data Fig. S4B). This suggests a highly converged group of integrators from different pathways in the regulation of floral timing and flower development of *C. sinense*. In the early stages, most of the genes were related to floral timing and meristem determinacy, while in the late stages there were hormonal regulators.

### Metabolomic analysis

**Figure 5:**
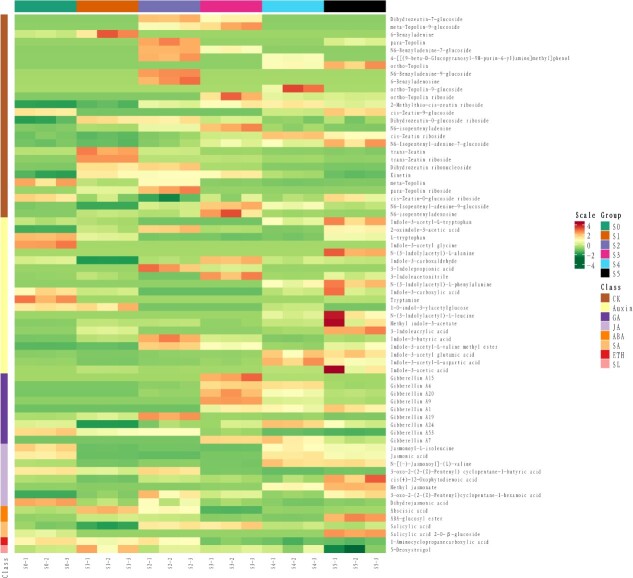
Metabolome of major plant hormones. The analysis was performed using UHLC. A total of 69 hormone-related metabolites were identified.

In line with transcriptome and proteome sequencing, the metabolome sequencing also generated a high-quality data set (Supplementary Data 1). The metabolome identified 69 potential chemicals associated with major plant hormones (Fig. 5). Cytokinins were the highest (26), followed by auxins (20), GAs (9), and jasmonic acids (8). Cytokinins were mainly active from middle stages of flower development (S1–S3). However, auxins did not show a clear pattern of variation among the six stages. Interestingly, GAs maintained an obvious concentration gradient across early and late stages of flower development. Most of the active GAs showed high concentration in the late stages after dormancy release, while only two inactive GAs showed high concentrations in the early dormant stages of flower development. Contrarily, ABA was high during dormant stages and maintained low levels during late stages.

### Gibberellin and abscisic acid signals are the key factors for *Cymbidium sinense* flower development

**Figure 6:**
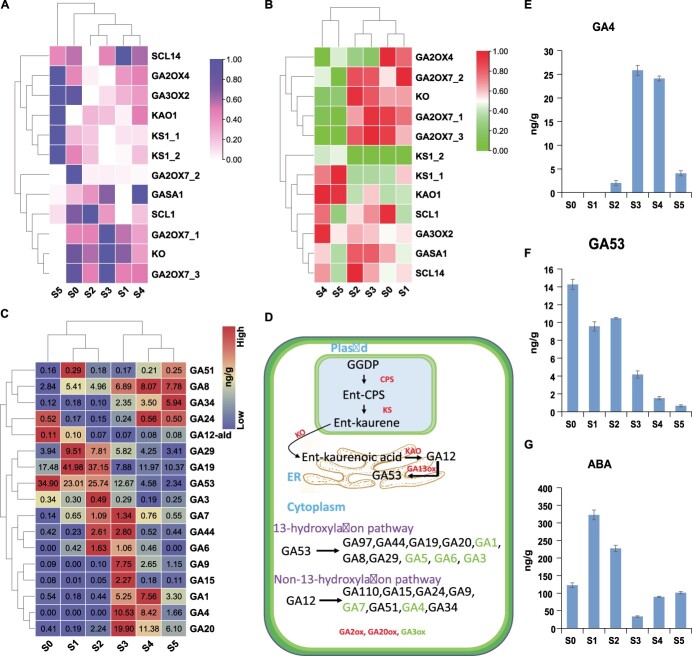
Overview of major gibberellins and pathway enzymes based on multi-omics. **A** Heat map showing the transcript levels of GA regulatory genes across six stages of flower development. **B** Heat map showing protein concentrations of GA pathway enzymes. **C** Heat map with concentration values of GAs identified by metabolomic analysis. Red color shows high values and blue shows low values. Concentrations are measured as nanograms per gram. **D** Depiction of GA pathway enzymes and production of major GAs within the cell. The process starts from plastids and then moves to the endoplasmic reticulum and final reactions occur in the cytoplasm. **E**, **F** Antagonistic role of GA4 and GA53 during axillary bud activation. **G** Concentration gradient of ABA found in the metabolomic analysis across six stages of flower development. Here ABA supports the role of GA53 during early stages of flower development.

We isolated 20 potential regulators of ABA and GA, including 11 ABA-related and 9 GA-related transcripts with corresponding protein concentrations (Supplementary Data Fig. S4A). Interestingly, during early stages of flower development, both the transcript and protein concentrations of GA and ABA were comparably opposite, suggesting their important role in bud dormancy and its release. The transcript levels of GA regulatory genes were considerably high in the late stages of flower development compared with early stages (Fig. 6A). The protein concentrations were high only for GA2ox genes during the early stages of flower development, while most of the other proteins showed high expression during the late stages (Fig. 6B). Consistent with this, the metabolome data also confirmed high amounts of most of the GAs during late stages of flower development except some inactive GAs during the early stages (Fig. 6C).

Most of the inactive GAs were abundant during early stages of flower development (Fig. 6C and D), while the active GAs were abundant during late stages. As a reference, we consider the antagonistic role of an active GA4 and an inactive GA53, as their amounts are significant compared with other hormones. The content of active GA4 is very low during dormancy, while the content of inactive GA53 is very high (Fig. 6E and F). Therefore, GA53 cannot be transformed into active GA4 during the first two stages of dormancy, and the key genes in this process are very important. Interestingly, just like GA53, the ABA level was also high during early dormant stages (Fig. 6G).

**Figure 7:**
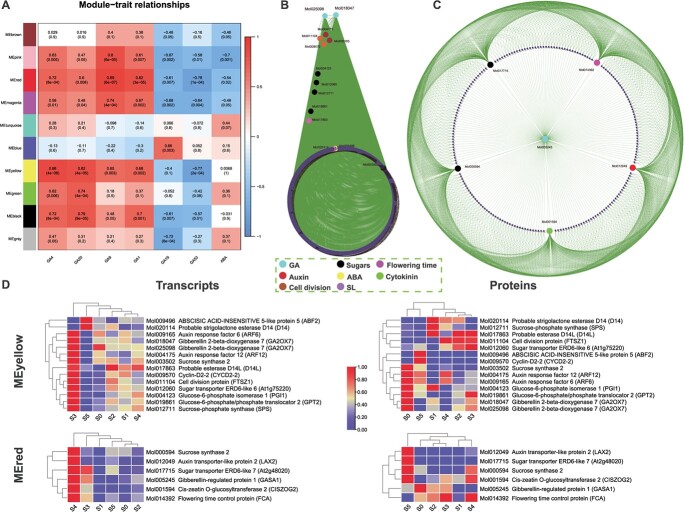
WGCNA-based correlation analysis of GAs and ABA and identification of hub genes. **A** Module–trait relationships. Six significantly differential GAs (GA1, GA4, GA9, GA19, GA20, and GA53) and ABA were used as traits based on metabolome data to draw their correlations with transcriptome data. Ten modules were identified. The columns denote hormones and the rows represent modules. Cell colors indicate correlation coefficients: blue for negative correlation and red for positive correlation. *P*-values are shown in brackets. MEyellow and MEred were found to be highly consistent with the opposite roles of GA4/GA1 and GA53/GA19. **B** Hub genes from the MEyellow module. From this module 14 hub genes were identified related to GA, auxin, cytokinin, sugar, cell cycle, flowering time, strigolactone, and ABA. **C** Hub genes from the MEred module. Here GA pathway genes connect all hub genes related to auxin, sugars, flowering time, and cytokinin. **D** Heat maps of transcript expressions and protein concentrations of the hub genes found in MEyellow and MEred.

Six significantly differential GAs (GA1, GA4, GA9, GA19, GA20, and GA53) and ABA were used based on metabolome data to infer their correlation with transcriptome data (Fig. 7A). Two highly significant modules, MEyellow and MEred, were found to be highly consistent with the opposite roles of GA4/GA1 and GA53/GA19. From MEyellow, we identified 14 hub genes related to GA, auxin, sugar, cell cycle, and ABA (Fig. 7B). The Cytoscape network showed the centrality of two GA genes. Similarly, the network from the MEred module showed a GA regulatory gene (GASA1) at the center connecting subnetworks formed by auxin, cytokinin, sugar, and flowering time regulatory genes (Fig. 7C). Most of the hub genes showed high transcript values in the late stages of flower development in both the modules (Fig. 7D). However, more than half of the proteins in the yellow modules showed high levels in S0 compared with other stages, while the proteins from MEred showed high levels in the late stages of flower development compared with early stages (Fig. 7D).

### Exogenous gibberellin triggers floral-related genes and promotes flower development

**Figure 8:**
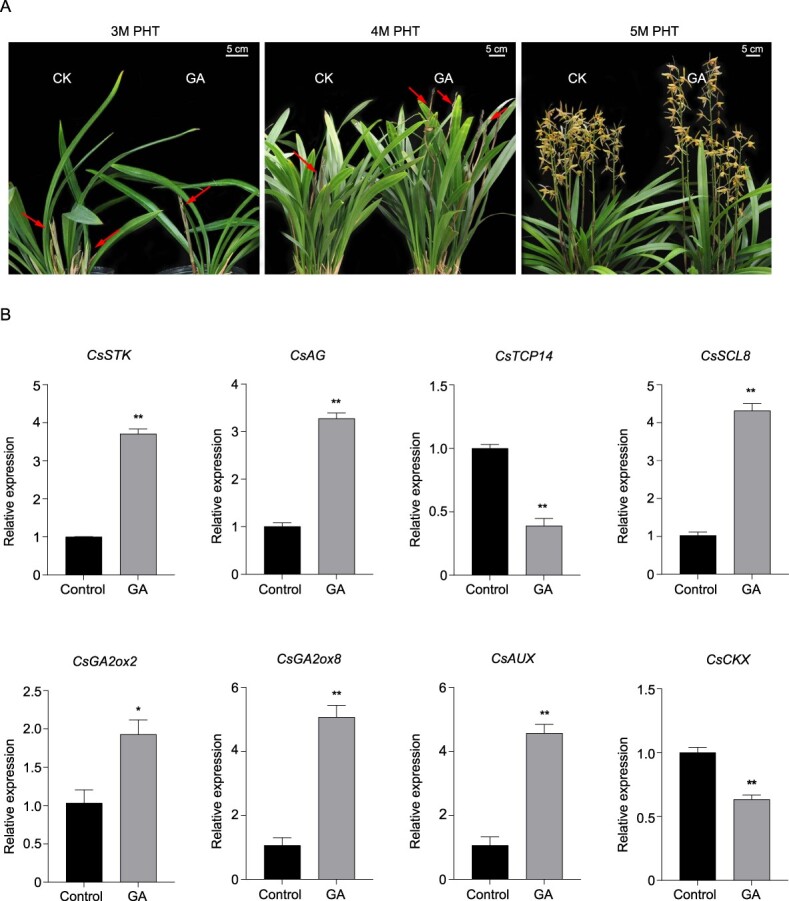
Effect on exogenous GA application on flower development and flowering pathway genes. **A** Effect of exogenous GA3 on floral bud and inflorescence characteristics. GA3 was applied at a concentration of 200 mg/l to 10 pots. Bud and inflorescence characteristics were recorded after 3 months of hormonal treatment. **B** Effect of exogenous GA application on expression of flowering and hormone-pathway genes.

Exogenous application of GA significantly promoted bud elongation. After 3 months of treatment with 200 mg/l GA, the length of the inflorescence axis was increased by 36.3% compared with the control group (Table 1; Fig. 8A). Moreover, GA application significantly increased the expression of flower development-related genes (*CsSTK* and *CsAG*), GA biosynthesis (*CsGA2ox2* and *CsGA2ox8*) and signaling (*CsSCL8*) genes, and auxin pathway genes, including *CsAUX*, *ARF*s, and *CsbZIP*s (Fig. 8B; Supplementary Data Fig. S5A). However, the expression of potentially negative regulatory factors, including *CsCKX* (cytokinin oxidase/dehydrogenase) and *CsTCP14*, was reduced after exogenous GA application.

**Table 1:** Effect of exogenous GA3 on floral bud and inflorescence characteristics of *C. sinense*.

**Treatment**	**1 M PHT**	**2 M PHT**	**3 M PHT**	**Mature scape length (cm)**	**Inflorescence length (cm)**	**Stem diameter (mm)**	**Flower diameter (cm)**
Control	5.87 ± 1.70	9.33 ± 3.06	40.83 ± 2.75	44.83 ± 0.76	15.67 ± 1.53	5.26 ± 1.37	4.75 ± 0.72
GA	5.90 ± 1.15	13.33 ± 4.16	55.67 ± 2.08^*^	58.50 ± 4.33^*^	15.33 ± 3.69	3.96 ± 0.67	5.21 ± 0.85

The first three columns show bud length (M PHT, months post hormonal treatment). ^*^P < 0.01.

### Abscisic acid inhibits flower development

Exogenous ABA application inhibited bud outgrowth (Table 2). However, after the application of ABA inhibitor, the elongation of flower branches was significantly advanced, reaching 37.37 ± 3.15 cm, although the difference was not significant at maturity. ABA inhibitor also affected flower area and inflorescence length.

**Table 2:** Effect of exogenous ABA and ABA inhibitor on the floral bud and inflorescence characteristics of *C. sinense*.

**Treatment**	**1 M PHT**	**2 M PHT**	**2.5 M PHT**	**Mature scape length (cm)**	**Inflorescence length (cm)**	**Stem diameter (mm)**	**Flower diameter (cm)**
Control	14.50 ± 1.32	24.97 ± 3.65	29.23 ± 3.37	49.23 ± 2.93	21.90 ± 1.39	5.71 ± 0.67	4.81 ± 0.62
ABA	15.23 ± 0.21	24.67 ± 3.82	27.80 ± 1.71^*^	46.67 ± 2.08	22.90 ± 1.82	5.39 ± 0.61	5.01 ± 0.44
ABA inhibitor	17.53 ± 2.16	31.83 ± 3.82	37.37 ± 3.15^*^	48.67 ± 3.06	19.07 ± 2.77	4.43 ± 0.46	3.67 ± 0.59

The first three columns show bud length. M PHT months post hormonal treatment. ^*^P < 0.01.

**Table 3:** Effect of exogenous ABA and ABA inhibitor on the flowering time and flower longevity of *C. sinense*.

**Treatment**	**Time to anthesis (days)**	**Flower longevity (days)**	**Inflorescence longevity (days)**
ABA	84.67 ± 1.53	12.33 ± 1.15^*^	24.67 ± 3.21
ABA inhibitor	76.50 ± 7.55^*^	16.00 ± 1.41	30.50 ± 4.12^*^
Control	84.60 ± 0.55	15.60 ± 2.70	25.80 ± 2.17

^*^ *P* < 0.05.

Time of anthesis was significantly reduced (76.50 ± 7.55 days) under the influence of ABA inhibitor compared with exogenous ABA (84.67 ± 1.53 days) and control (84.60 ± 0.55 days) (Table 3). A significant flower longevity of 16.00 ± 1.41 days was observed due to ABA inhibitor, flowers lasting for 4 days longer than exogenous ABA-treated flowers. Similarly, inflorescence longevity was potentially high (30.50 ± 4.12 days) in the axillary branches treated with ABA inhibitor (Table 3).

### Abscisic acid-responsive *CsAP1* regulates flower development and inflorescence structure

**Figure 9:**
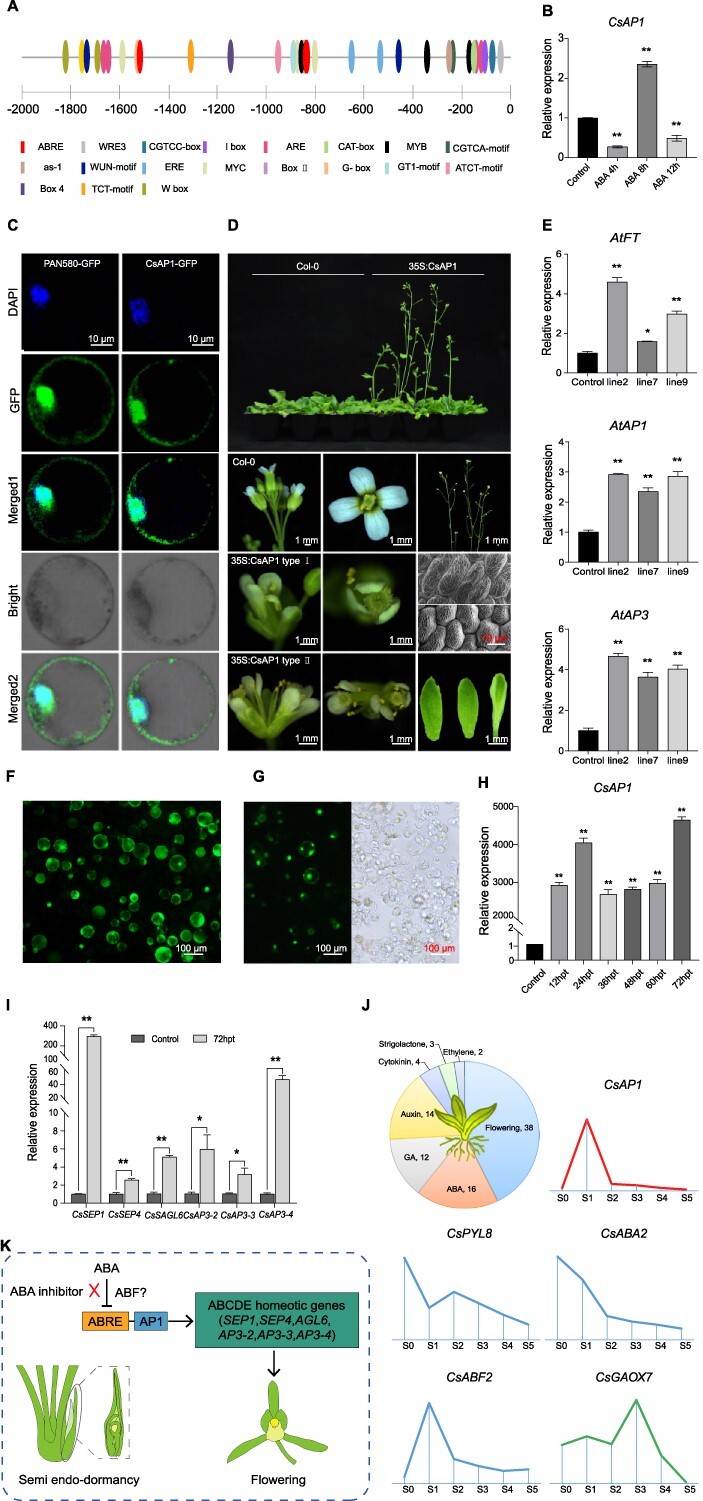
Relationship between ABA and AP1 towards the regulation of flower bud initiation and development. **A***Cis*-element analysis of *CsAP1* promoter. The promoter sequence of the *CsAP1* gene was acquired from the reference *C. sinense* genome. Primers were designed and the full-length *CsAP1* promoter was obtained by PCR. The *cis*-acting elements of the *CsAP1* promoter were predicted using the online software PlantCARE (http://bioinformatics.psb.ugent.be/webtools/plantcare/html/). **B** Expression level of *CsAP1* under exogenous ABA treatment in *C. sinense*. **C** Subcellular localization analysis of *CsAP1*. **D** Overexpression of CsAP1 in *Arabidopsis*. The *CsAP1* gene was cloned and transformed into *Arabidopsis* by *Agrobacterium*-mediated transformation. Phenotypic data were recorded on flowering time and flower organ shapes and structures in *CsAP1*-transgenic lines. Scanning electron microscopy (bottom right panels) was performed to check sepal and petal epidermis of transgenic *Arabidopsis*: upper panel show sepal epidermal cells and lower panel shows petal epidermal cells. **E** qRT–PCR results of *Arabidopsis thaliana CsAP1* transgenic lines. Expression of *AtFT*, *AtAP1*, and *AtAP3* was checked after overexpression of *CsAP1*. **F** Transient overexpression of *CsAP1* in the protoplast of *C. sinense* and measurement of protoplast viability using the FDA staining method. **G** Transfection efficiency of *CsAP1* after transient overexpression in the protoplast of *C. sinense*. **H** qRT–PCR results of transiently overexpressed *CsAP1* in *C. sinense* protoplasts. **I** qRT–PCR expression of flowering pathway genes after transient overexpression of *CsAP1* in the protoplast of *C. sinense*. **J** Summary of transcriptome data emphasizing the importance of hormones and the relationships among *CsAP1*, ABA, and GA. **K** Proposed pathway of floral bud regulation through the interaction of ABA and AP1. AP1 promotes bud activation by upregulating several genes from the ABCDE model. ABA may inhibit AP1 function by acting on the ABA response element (ABRE) present in the promoter region of AP1. ABA inhibitor can release this effect by countering the inhibitory effect of ABA on ABRE. However, how ABF (ABRE-binding factor) controls this binding needs further research.

To reveal the regulatory function of ABA in flower bud development, we compared the transcriptome of flower buds before and after treatment with ABA and its inhibitors and screened for differentially expressed genes (DEGs). Among them, *CsAP1* and its coexpressed genes related to flower development and/or hormone signaling were found to have the most significant differential expression changes. This result prompted us to test the function of *CsAP1*. *In silico* promoter analysis indicated potential ABA response elements (ABREs) located in the 2000 bp upstream of ATG (stop codon) (Fig. 9A). Floral buds were treated with exogenous ABA and the response of *CsAP1* was observed. The expression level first decreased 4 h after treatment and a significant increase was observed after 5 h, and then it decreased again after 12 h (Fig. 9B). Subcellular localization analysis of *CsAP1* indicated bright signals in the membrane and nucleus (Fig. 9C).

We overexpressed *CsAP1* in *Arabidopsis* (Supplementary Data Fig. S5B) and found that *CsAP1* impacted flower organ morphology and inflorescence structure (Fig. 9D). In addition to an early flowering phenotype, the flower sepals and petals also showed modifications. Moreover, compound terminal flowers were observed in *35S:CsAP1* plants containing three or four pistils with abnormal numbers of sepals, petals, and stamens. Scanning electron microscopy of epidermal cells of sepal and petal further confirmed the abnormality in the arrangement of cells (Fig. 9D). We ascertained the expression of several key regulators of flowering- and flower development-related genes and found a significant increase in their expression (Fig. 9E; Supplementary Data Fig. S5C).

To further verify the role of *CsAP1* in *C. sinense*, we transiently overexpressed *CsAP1* in the protoplast of *C. sinense* (Fig. 9F and G) and observed significantly high expression until 72 h (Fig. 9H). Overexpression of *CsAP1* in *C. sinense* induced downstream flowering-related genes, including *SEP1*, *SEP4*, *AGL6*, *AP3-2*, *AP3-3*, and *AP3-4* (Fig. 9I). Our transcriptome data also support the reciprocal relationship between *CsAP1* and ABA (Fig. 9J). From an abundant array of floral and hormonal regulators, *CsAP1* expression was well aligned with GA but opposite to ABA through different stages of flower development.

We thus proposed a molecular regulatory model of flower development mediated by the ABA signaling pathway (Fig. 9K). In normal cases ABA can counter the effect of *CsAP1* using its response element ABRE, which ultimately affects the function of floral homeotic genes in the ABCDE model, including *SEP*s, *AGL6* and *AP3*s. However, ABA inhibitor can release the inhibitory effect of ABA and break the semi-endodormancy of the *C. sinense* floral bud (Fig. 9K).

## Discussion

As perennial herbs, most orchids bloom under specific conditions. For most seasonal flowering plants, it takes several months from bud differentiation to flower opening, and a period of bud (semi)dormancy is needed. During cold conditions, bud dormancy prevails and the DAM genes emerge as pivotal regulators of this dormancy process [8, 9]. However, no DAM orthologs are found in *C. sinense* [3], pointing to a different plan in orchids compared with perennial species using the SVP/StMADS11-regulated network [8, 9]. A recent study in peach blossom shows that it is different from other Rosaceae plants in the regulation of low-temperature flowering, indicating that the mechanism of flowering regulation is species-specific [41]. Why flower buds cannot grow continuously, needing a dormancy pause to continue growth and blooming, has always been an unsolved mystery in orchid plants.

The *Cymbidium* orchids produce floral buds from the leaf axils and their process of floral development, from initiation to flower opening, takes ~6–7 months. Compared with continuous flowering cultivars, the development of the lip and column of seasonal flowering *C. sinense* remains suspended (~3 months). Moreover, the labellum does not develop during the dormancy stages, indicating that the genes controlling the development of petals and labellum are different, and the differentiation and development of flower organs are not synchronized. Following the changes in floral developmental stages over time (Fig. 1), the comparative transcriptome and proteome data showed stage-specific gene clusters (Fig. 2B). *LOX*, *Hd3a*, and *HDT2* were specifically expressed in the early floral development stages (FD0–FD2). These genes play significant roles in the vegetative to reproductive phase transition and cell division [42–44]. However, the genes particularly expressed in the late stages of flower development (FD3–FD5) were mainly related to the energy production system, flavonoid pathways, and fatty acid metabolism (Supplementary Data Fig. S3C). The key hubs found in WGCNA were related to flowering and hormonal regulation (Fig. 4), suggesting the enrichment of genetic information during flower initiation and development. Among the hormone-related DEGs, auxins, GAs and ABAs were the most abundant genes (Supplementary Data Fig. S4A).

Auxin has long been implicated in numerous aspects of plant growth and development, especially apical dominance. Conversion of an inflorescence meristem to floral meristem requires normal polar auxin transport [45]. Auxin response factors and auxin-binding proteins were abundant among the auxin-related DEGs (Supplementary Data Fig. S4A). The metabolomic data also contained 26 auxins which were unevenly distributed among six stages of flower development (Fig. 5). However, we did not find a certain pattern or homogeneity among transcriptomic, proteomic, and metabolomic data regarding auxin regulation. Therefore, we investigated GA and ABA, the two most abundant hormones after auxin found in our data.

Axillary buds are regulated by a network of promotive and inhibitory forces. Our multi-omics results show that specific GAs promote branching, while others maintain the dormancy of axillary buds. Especially, the antagonistic role of GA4 and GA53 is crucial to maintain different phases of axillary bud growth (Fig. 6). In hybrid aspen, significantly high expression of *GA20ox* genes was observed in the axillary buds compared with apices and the levels of bioactive GA1/4 were significantly lower in axillary buds than the proliferating apices [11]. During 2β-hydroxylation, the encoded GA2ox enzymes irreversibly deactivate the bioactive GAs and therefore high expression of *GA2ox* can keep axillary buds quiescent [11, 46, 47]. GA4 plays a significant role in energy metabolism, cell division, and elongation [48, 49]. Maintaining a low level of GA4 is necessary to inhibit the activation and outgrowth of axillary buds [11]. In line with these suppositions, our results showed low concentrations of GA4 during early stages of flower development, supporting bud dormancy (Fig. 6E), while the inactive GA53 was significantly high during early stages (Fig. 6F). Therefore, significant reduction of GA deactivation enhances the bioactive GA1/4 pool to spearhead the activation of axillary buds.

Correlation between GAs and transcriptome data suggested two potential modules (MEyellow and MEred) with contrasting correlation coefficients for GA4/1 and GA53/19 (Fig. 7A). Both the modules contained GA pathway genes as hubs connecting multiple subnetworks formed by important flowering and hormonal regulators (Fig. 7B and C). We can therefore suggest that GA has an integral role in the regulation of bud dormancy and bud activation of *C. sinense*. However, extensive functional research is required to fully disentangle this complex regulatory conduit.

ABA is an essential plant hormone that modulates various physiological and molecular mechanisms during axillary bud dormancy, its activation and development. ABA inhibitor significantly triggered *C. sinense* floral buds (Table 2) and advanced the time to anthesis (Table 3) compared with exogenous ABA application. Artificial application of ABA increased the flowering rate and advanced flower formation in apple [50]. However, the role of ABA remains controversial as researchers have reported both positive and negative effects of ABA on the floral transition [20, 51, 52]. ABA is triggered by short days and it interrupts intercellular communication in buds, which promotes endodormancy. Additionally, exposure to short days also induces the expression of *SVL* [*SVP* (*SHORT VEGETATIVE PHASE*)*-LIKE*], which establishes a positive feedback loop with ABA [53]. *SVL* upregulates CALS1 (callose synthase), which blocks plasmodesmata with dormancy sphincters (callosic plugs), thereby obstructing intercellular communication and slowing down cell activities in buds [54]. *SVL* also promotes bud dormancy by *BRC1* (*BRANCHED 1*)-mediated inhibition of *FT* [55]. *LAP1* (*Arabidopsis* ortholog of *AP1*) negatively regulates *BRC1*, thus forming a negative feedback loop that regulates seasonal growth through FT [55]. Exposing plants to short-term cold temperature increases the concentration of ABA, leading to the upregulation of *DAM*/*SVL* and consequently triggering bud dormancy [56]. In poplar, the expression of *EBB1* (*EARLY BUD-BREAK1*), a transcription factor belonging to the APETALA 2 (AP2) family, is upregulated in response to low temperatures. This upregulation leads to the suppression of *SVL* expression, which breaks the SVL/ABA feedforward loop, resulting in the release of bud dormancy [57].

*APETALA1* (*AP1*) is a floral meristem identity gene that plays a crucial role in specifying floral meristem during floral transition [23]. Emerging meristem requires *AP1* to partly specify its floral identity through direct repression of flowering time genes, including *SOC1* (*SUPPRESSION OF OVEREXPRESSION OF CO1*), *SVP*, and *AGL24* (*AGAMOUS-LIKE24*) [23]. *AP1*-homologous genes function in flower development, with functional differentiation in different species. *AP1* plays a key role during the transition phase from flower induction to flower formation by acting as a switch between the two phases and constructing a hub in the corresponding gene regulatory network [58]. During early stages of flower development, *AP1* acts as a transcriptional repressor; however, at more advanced stages it involves floral organ formation by activating the regulatory genes, indicating its dynamic roles. Overexpression of *CsAP1* in *Arabidopsis* instigated early flowering, modifications in the sepals and petals, and compound terminal flowers containing three or four pistils with abnormal numbers of sepals, petals, and stamens (Fig. 9D). Moreover, it significantly increased the expression of key floral integrators, including *FT*, *AP1*, and *AP3* (Fig. 9E). This suggests that *CsAP1* has a major role in flower initiation and organogenesis. Exogenous ABA application enhanced the expression of *LcAP1* in litchi, whereas ABA inhibitor suppressed its expression [22], suggesting that ABA can regulate flowering through *AP1*. However, the situation is opposite in *C. sinense*. We observed a considerably low expression of *CsAP1* after exogenous ABA application (Fig. 9B), indicating that homologous genes have different responses to external environmental signals, species-specific molecular mechanisms probably exist. The presence of ABRE in the promoter region of flowering-related genes enables the specific binding of ABRE-binding protein/ABRE-binding factor (AREB/ABF) [59], which serves as a regulatory mechanism to control the expression of these genes in response to ABA. ABRE-mediated transcription of downstream target genes is regulated by AREB/ABF [60]. By modulating the expression of these genes, ABA can fine-tune the flowering process in plants. Therefore, we propose that ABA inhibitor may release bud dormancy by repressing the ABRE, thereby allowing proper functioning of *CsAP1* and bud outgrowth (Fig. 9K). In support of this proposition, the transcriptome data exhibited a reciprocal relationship between *CsAP1* and ABA (Fig. 9J). Considering bud dormancy regulation, the ABA–*CsAP1* loop is of prime importance in *Cymbidium* orchids. However, how ABF facilitates specific functioning of ABRE and how its role is species-specific need further research.

## Materials and methods

### Plant materials and growth conditions

The *C. sinense* plants were cultivated in the greenhouse facility of the Institute of Environmental Horticulture (Guangdong Academy of Agricultural Sciences, China). The growth conditions were set to a day/night temperature of 25/20°C with a photoperiod of 18/8 h. Samples were collected in three replicates from six stages of flower development, FD0–FD5. Samples were immediately stored at −80°C until RNA extraction.

### RNA sequencing library preparation and transcriptome sequencing

RNA was extracted using a TaKaRa RNA extraction kit and cDNA libraries were produced, followed by RNA filtering using an Oligotex mRNA Midi Kit (Qiagen, Germany). RNA quality and quantity were checked on a NanoDrop 2000 spectrophotometer (Thermo Scientific, USA) and cDNA libraries were prepared following the Illumina protocol. The purified library products were evaluated using the Agilent 2200 TapeStation and Qubit^®^ 2.0 (Life Technologies, USA). The products were diluted to 10 pM for the generation of *in situ* clusters on the HiSeq 2500 paired-end flow cell and paired-end sequencing (2 × 100 bp) was performed. Transcriptome *de novo* assembly was performed using the Trinity program with default parameters [61].

### Proteome profiling

Protein extraction and subsequence analyses and proteome profiling were achieved following the method described by Guo *et al*. [62].

### Metabolomic analysis

The floral metabolome was detected using ultra-high-performance liquid chromatography (UHLC) as previously described [63].

### Identification of differentially expressed genes and annotation analyses

Gene expression levels were ascertained using FPKM values. The edgeR package was used to identify DEGs at FDR < 0.05 and log_2_ ratio > 1 (2-fold change). The correlation among the samples was determined using the Pearson correlation coefficient and PCA. GO annotation was performed using InterProScan (v.5.14–53.0; http://www.ebi.ac.uk/interpro/), and KAAS (v.2.0; http://www.genome.jp/kaas-bin/kaas_main) and KEGG Mapper (v.2.5; http://www.kegg.jp/kegg/mapper.html) were used for KEGG analysis. The Perl module (v.1.31; https://metacpan.org/pod/Text::NSP::Measures::2D::Fisher) was used for enrichment analyses and the R package pheatmap was used to produce various cluster heat maps (v.2.0.3; https://cran.r-project.org/web/packages/cluster/).

### Gene set enrichment and weighted gene coexpression network analyses

GSEA was performed based on KEGG pathways to reveal the pathways involved in proteins or transcripts under different regulatory relationships.

For WGCNA, the transcripts were selected with corresponding protein concentrations. A total of 6905 transcripts were run in the WGCNA analysis as previously described [64]. After the removal of bad genes with poor expression and low connectivity, 5553 genes were selected for module construction. The Cytoscape edges of selected modules were run in Cytoscape (v3.9.1) and the hub genes were selected using the cytoHubba application in Cytoscape.

### Genetic transformation of *Arabidopsis*

Wild *Arabidopsis* plants were obtained from seeds grown on a mix of peat soil and coconut bran, mixed with ¼ MS culture solution. The transformation was performed at the time of blooming. The *CsAP1* gene was cloned (Supplementary Data Table S4) and transformed into *Arabidopsis* by *Agrobacterium*-mediated transformation [65], using the floral dip method. The seeds were harvested at maturity.

The infected seeds were grown on ½ MS medium plates (containing 50 mg/l hygromycin) at a vernalization temperature of 4°C for 2–3 days. After vernalization, the plates were moved to the tissue culture room for cultivation, and at the two-cotyledon stage the seedlings were transferred to small pots (6–8 cm) and placed in an artificial climate incubator for cultivation until blooming.

### Real-time quantitative PCR to identify the expression level of transgenic plants

RNA extraction of transgenic *Arabidopsis* leaf was performed using a Tiangen RNA extraction kit, followed by reverse transcription into cDNA. *Arabidopsis* actin was used as an internal reference for amplification on a real-time PCR instrument. Using wild-type *Arabidopsis* as a control, the gene expression levels of the overexpressed lines were determined.

### Plant growth regulator treatments

The experiment was set up with plant growth regulator treatments (GA, ABA, and ABA inhibitor) and clear water treatment as the control. The hormone solutions included 200 mg/l GA, 100 mg/l ABA, or 5 mmol/l ABA inhibitor. Each treatment was applied to 10 pots and repeated three times, a total of 30 pots. The first experiment started on 29 October 2021. At this time the flower bud was ~4.78 cm long, which was in the second stage of flower bud differentiation and development. After plant growth regulator treatment, sampling was done for expression studies.

### Protoplast-mediated transient expression assay of CsAP1-1

The protocol for transient expression in the protoplast of *C. sinense* has been described previously [66]. Briefly, the vector for transient expression was constructed by inserting the CDS sequence of CsAP1-1 into the PAN580-GFP vector. Primers specific to CsAP1-1 were designed with overlapping homologous ends using Primer Premier 5.0 (Premier, USA) using the full-length AP1-1 CDS (Supplementary Data Table S4). Then, the gene fragments were cloned into PAN580-GFP vector following the Seamless Assembly Cloning Kit (CloneSmarter, USA) according to the manufacturer’s protocol. The recombined vectors were transferred into *Escherichia coli* DH5α-competent cells (Tiangen, China) and the transformation was confirmed by sequencing. The bacteria were replicated, and plasmid DNA was extracted using an Endo-Free Plasmid Maxi Kit (Omega Bio-tek, USA). Concentrated (2.0 μg/μl) plasmid DNA was used for protoplast transfection.

The PEG-mediated protocol was used with little modifications [67] for protoplast transfection. Briefly, the PEG solution [PEG 4000 (40% w/v), 0.1 M CaCl_2_, and 0.2 M mannitol] was mixed with plasmid DNA in MMG solution [4 Mm MES (pH = 5.7), 15 mM MgCl_2_, and 0.4 M mannitol]. The transfected protoplasts were incubated in the dark at 23°C for 6–24 h. Transient expression was measured according to the GFP reporter expression of Pan580-GFP vector. GFP fluorescence was measured using a LSM710 confocal laser scanning microscope.

### qRT–PCR expression of transient CsAP1-1 and other genes

Protoplasts with transient CsAP1-1 were harvested at 12, 24, 36, 48, 60 and 72 h after transmission. Primers were designed for APs, ARFs, SEPs, AGL6, FT, FRI, and SOC1, according to their CDS sequences using Primer Premier 5.0 (Supplementary Data Table S5). *CsUBQ* was used as an internal standard. qRT–PCR was performed in a 20-μl reaction mixture containing 2.0 μl of first-strand cDNA, 10.0 μl of 2 × SYBR Green I Master Mix (Takara, China), 0.8 μl of each primer, and 6.4 μl of sterile distilled water. The reactions were performed using a CFX-96 Real-Time PCR System (Bio-Rad, USA). The following conditions were applied: starting 5-min cycle at 95°C, 40 cycles at 95°C for 15 s, and 60°C for 30 s, followed by 72°C for 30 s, and a final 5-min cycle at 68°C. The relative quantification method (2^−ΔΔΤ^) was used to quantify gene expression [66].

### Promoter isolation and analysis

DNA was extracted from floral buds using the CTAB method and the promoter sequence (Supplementary Data Table S4) of the CsAP1 gene was acquired from the reference *C. sinense* genome [3]. Primers were designed (Supplementary Data Table S5) and the full-length CsAP1 promoter (Supplementary Data Table S4) was obtained by PCR. Finally, a 2000-bp long promoter sequence was acquired. The *cis*-acting elements of the CsAP1 promoter were predicted using the online software PlantCARE (http://bioinformatics.psb.ugent.be/webtools/plantcare/html/).

### Subcellular localization of CsAP1

The CsAP1 coding sequence without stop codon was cloned into pan580-green fluorescent protein (GFP), resulting in fusion protein CsAP1–GFP. Protoplast-based transient expression was employed to achieve the transformation of plasmid DNA into protoplasts of *C. sinense*. For the detection of nuclei, the transfected protoplasts were stained with 50 μg/ml DAPI (4′-6′-diamidino-2-phenylindole) at 37°C for 10 min. UV light was used to illuminate DAPI in fluorescence microscopy.

### Scanning electron microscopy

The collection of lateral buds was performed every week (20 June to 1 September). The outer scales on buds were removed and buds were fixed using a fixation solution (2% formaldehyde and 3% glutaraldehyde) for 24 h. After fixation, dehydration was performed using acetone, followed by critical-point drying in liquid CO_2_. Finally, the samples were mounted on stubs and sputter-coated with 25nm gold. A JSM-6360LV (JEOL) scanning electron microscope was used to visualize the samples.

### Statistical analysis

One-way ANOVA was used on SPSS software (Inc., Chicago, USA; v.16.0) to calculate significant differences Significance is shown in the tables and figures as ** for *P* < 0.01 and * for *P* < 0.05.

## Supplementary Material

Web_Material_uhae073

## Data Availability

The original contributions presented in the study are publicly available. The supplementary data are provided along with the manuscript as supplementary tables and supplementary figures. The transcriptome sequencing clean data have been uploaded to the database of the BIG Data Center (http://gsa.big.ac.cn/index.jsp) under accession number PRJCA025438. Proteome data are available via ProteomeXchange with identifier PXD051591.
