# Supplementary material for: Integrated proteomic, transcriptomic, and metabolomic profiling reveals that the gibberellin–abscisic acid hub runs flower development in the Chinese orchid Cymbidium sinense
Source: Hortic Res. 2024 Mar 12;11(5):uhae073. doi: 10.1093/hr/uhae073 (PMC11088716; doi:10.1093/hr/uhae073)
Supplement: Web_Material_uhae073 [file web_material_uhae073.zip › Supplementary data 1.docx]

1. **Quality evaluation of transcriptome data**

**Overview of sequencing data quality control**

| Sample | Raw_Reads | Raw_Bases | Valid_Reads | Valid_Bases | Valid% | Q20% | Q30% | GC% |
| --- | --- | --- | --- | --- | --- | --- | --- | --- |
| S0_1 | 53917590 | 8.09G | 52154748 | 7.82G | 96.73 | 99.98 | 97.56 | 45 |
| S0_2 | 51129994 | 7.67G | 49866996 | 7.48G | 97.53 | 99.98 | 97.48 | 45 |
| S0_3 | 53990210 | 8.10G | 52187778 | 7.83G | 96.66 | 99.98 | 97.45 | 45 |
| S1_1 | 70540398 | 10.65G | 69411148 | 10.33G | 98.4 | 98.81 | 96.52 | 44.74 |
| S1_2 | 70968132 | 10.72G | 69914198 | 10.41G | 98.51 | 98.84 | 96.56 | 46.41 |
| S1_3 | 70632958 | 10.67G | 69528412 | 10.35G | 98.44 | 98.82 | 96.54 | 46.41 |
| S2_1 | 69756098 | 10.53G | 68686684 | 10.22G | 98.47 | 98.83 | 96.55 | 45.13 |
| S2_2 | 72838588 | 11.00G | 71720382 | 10.67G | 98.46 | 98.82 | 96.56 | 45.72 |
| S2_3 | 66255590 | 10.00G | 65235140 | 9.70G | 98.46 | 98.79 | 96.47 | 45.53 |
| S3_1 | 67498830 | 10.19G | 66382074 | 9.88G | 98.35 | 98.89 | 96.66 | 45.4 |
| S3_2 | 69875294 | 10.55G | 68763398 | 10.23G | 98.41 | 98.9 | 96.72 | 44.36 |
| S3_3 | 69692766 | 10.52G | 68584484 | 10.21G | 98.41 | 98.91 | 96.7 | 45.32 |
| S4_1 | 67571336 | 10.20G | 66512996 | 9.90G | 98.43 | 98.87 | 96.59 | 46.21 |
| S4_2 | 71659436 | 10.82G | 70516768 | 10.50G | 98.41 | 98.93 | 96.73 | 46.48 |
| S4_3 | 71617828 | 10.81G | 70363064 | 10.47G | 98.25 | 98.84 | 96.52 | 46.15 |
| S5_1 | 67135962 | 10.14G | 66094042 | 9.84G | 98.45 | 98.89 | 96.64 | 46.31 |
| S5_2 | 70663018 | 10.67G | 69466736 | 10.34G | 98.31 | 98.87 | 96.58 | 46.46 |
| S5_3 | 71586476 | 10.81G | 70404564 | 10.48G | 98.35 | 98.9 | 96.68 | 45.73 |

#### Reference genome comparison Reads statistics

| Sample | Valid reads | Mapped reads | Unique Mapped reads | Multi Mapped reads | PE Mapped reads | Reads map to sense strand | Reads map to antisense strand | Non-splice reads | Splice reads |
| --- | --- | --- | --- | --- | --- | --- | --- | --- | --- |
| S1_1 | 69411148 | 62313962(89.78%) | 40473756(58.31%) | 21840206(31.46%) | 56866500(81.93%) | 30236509(43.56%) | 30154755(43.44%) | 36383654(52.42%) | 24007610(34.59%) |
| S1_2 | 69914198 | 51174129(73.20%) | 33321316(47.66%) | 17852813(25.54%) | 46719536(66.82%) | 24857642(35.55%) | 24795749(35.47%) | 30130412(43.10%) | 19522979(27.92%) |
| S1_3 | 69528412 | 55543041(79.89%) | 36105878(51.93%) | 19437163(27.96%) | 50564244(72.72%) | 26973191(38.79%) | 26907223(38.70%) | 33011871(47.48%) | 20868543(30.01%) |
| S2_1 | 68686684 | 59325850(86.37%) | 38202623(55.62%) | 21123227(30.75%) | 53118138(77.33%) | 28659340(41.72%) | 28622113(41.67%) | 34218439(49.82%) | 23063014(33.58%) |
| S2_2 | 71720382 | 58295799(81.28%) | 37307676(52.02%) | 20988123(29.26%) | 52284488(72.90%) | 28214723(39.34%) | 28135920(39.23%) | 34204502(47.69%) | 22146141(30.88%) |
| S2_3 | 65235140 | 54467762(83.49%) | 34968416(53.60%) | 19499346(29.89%) | 48457362(74.28%) | 26341945(40.38%) | 26279732(40.28%) | 31959859(48.99%) | 20661818(31.67%) |
| S3_1 | 66382074 | 60875319(91.70%) | 39994543(60.25%) | 20880776(31.46%) | 55537086(83.66%) | 29412769(44.31%) | 29339929(44.20%) | 33790064(50.90%) | 24962634(37.60%) |
| S3_2 | 68763398 | 62465178(90.84%) | 41019914(59.65%) | 21445264(31.19%) | 56923434(82.78%) | 30195932(43.91%) | 30128209(43.81%) | 34952155(50.83%) | 25371986(36.90%) |
| S3_3 | 68584484 | 62673310(91.38%) | 41039706(59.84%) | 21633604(31.54%) | 57142816(83.32%) | 30303689(44.18%) | 30230175(44.08%) | 35389329(51.60%) | 25144535(36.66%) |
| S4_1 | 66512996 | 60698436(91.26%) | 39651958(59.62%) | 21046478(31.64%) | 54744650(82.31%) | 29304267(44.06%) | 29237693(43.96%) | 36109238(54.29%) | 22432722(33.73%) |
| S4_2 | 70516768 | 64810949(91.91%) | 42186600(59.82%) | 22624349(32.08%) | 58853194(83.46%) | 31228266(44.28%) | 31208229(44.26%) | 37562745(53.27%) | 24873750(35.27%) |
| S4_3 | 70363064 | 64300607(91.38%) | 41623398(59.16%) | 22677209(32.23%) | 58216954(82.74%) | 31036249(44.11%) | 30982258(44.03%) | 37623067(53.47%) | 24395440(34.67%) |
| S5_1 | 66094042 | 60425656(91.42%) | 38176604(57.76%) | 22249052(33.66%) | 54377758(82.27%) | 29098657(44.03%) | 28887623(43.71%) | 34156230(51.68%) | 23830050(36.05%) |
| S5_2 | 69466736 | 63189194(90.96%) | 39462911(56.81%) | 23726283(34.15%) | 56197330(80.90%) | 30306352(43.63%) | 30126093(43.37%) | 36356106(52.34%) | 24076339(34.66%) |
| S5_3 | 70404564 | 64085375(91.02%) | 40187243(57.08%) | 23898132(33.94%) | 57474122(81.63%) | 30830172(43.79%) | 30621117(43.49%) | 36342988(51.62%) | 25108301(35.66%) |
| S0_1 | 52154748 | 37772549(72.42%) | 27150689(52.06%) | 10621860(20.37%) | 30889320(59.23%) | 18131627(34.77%) | 18178021(34.85%) | 23612964(45.27%) | 12696684(24.34%) |
| S0_2 | 49866996 | 31136908(62.44%) | 22187678(44.49%) | 8949230(17.95%) | 26260806(52.66%) | 14961786(30.00%) | 14993765(30.07%) | 19335888(38.77%) | 10619663(21.30%) |
| S0_3 | 52187778 | 33318430(63.84%) | 23761927(45.53%) | 9556503(18.31%) | 27641198(52.96%) | 16011612(30.68%) | 16049344(30.75%) | 20551599(39.38%) | 11509357(22.05%) |

**Distribution statistics table of gene expression values ​​for each sample**

| **Sample** | **Exp gene** | **Min.** | **1st Qu.** | **Median** | **Mean** | **3rd Qu.** | **Max.** | **Sd.** | **Sum.** |
| --- | --- | --- | --- | --- | --- | --- | --- | --- | --- |
| S1_1 | 35551 | 0.00 | 0.02 | 2.78 | 51.08 | 29.94 | 12139.12 | 198.39 | 1816005.82 |
| S1_2 | 35551 | 0.00 | 0.01 | 2.61 | 50.79 | 28.84 | 13914.38 | 215.81 | 1805653.88 |
| S1_3 | 35551 | 0.00 | 0.02 | 2.68 | 50.89 | 29.44 | 13406.79 | 213.55 | 1809233.82 |
| S2_1 | 35551 | 0.00 | 0.03 | 2.93 | 50.95 | 29.96 | 10147.59 | 197.49 | 1811446.25 |
| S2_2 | 35551 | 0.00 | 0.03 | 2.99 | 50.42 | 29.93 | 12095.28 | 204.73 | 1792483.16 |
| S2_3 | 35551 | 0.00 | 0.03 | 3.02 | 50.58 | 29.95 | 9914.29 | 199.28 | 1798190.36 |
| S3_1 | 35551 | 0.00 | 0.03 | 2.74 | 51.24 | 30.26 | 9709.01 | 208.23 | 1821505.54 |
| S3_2 | 35551 | 0.00 | 0.02 | 2.62 | 51.19 | 29.32 | 13776.17 | 209.47 | 1819684.78 |
| S3_3 | 35551 | 0.00 | 0.03 | 2.79 | 51.01 | 29.85 | 14124.35 | 214.59 | 1813455.30 |
| S4_1 | 35551 | 0.00 | 0.04 | 2.88 | 49.65 | 28.21 | 20441.28 | 234.33 | 1765178.06 |
| S4_2 | 35551 | 0.00 | 0.02 | 1.97 | 49.54 | 24.12 | 26342.22 | 294.26 | 1761254.09 |
| S4_3 | 35551 | 0.00 | 0.04 | 2.72 | 49.78 | 27.23 | 20197.18 | 249.98 | 1769628.49 |
| S5_1 | 35551 | 0.00 | 0.04 | 2.37 | 48.63 | 24.40 | 42087.74 | 398.75 | 1728707.52 |

1. **Quality evaluation of proteome data**


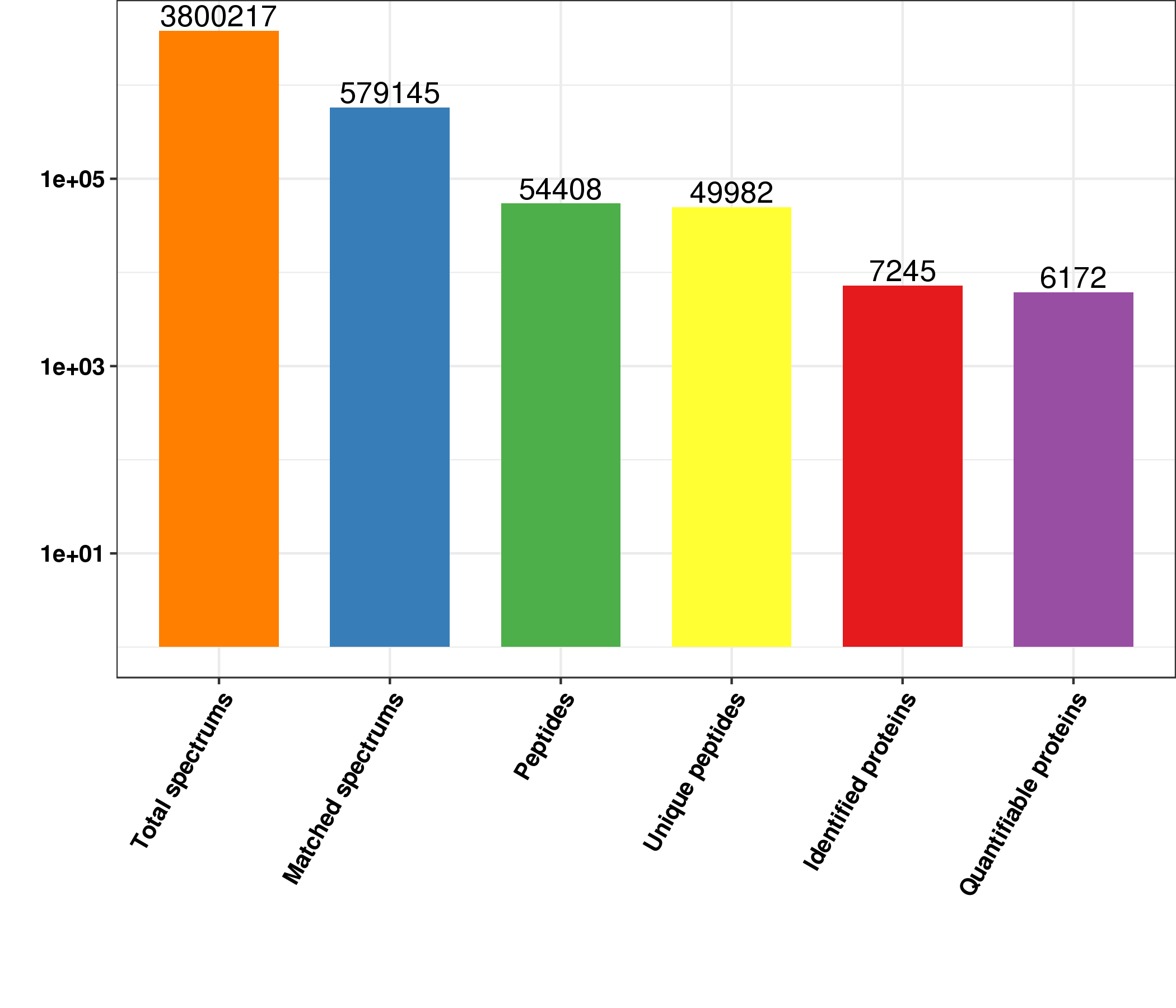


**Figure 1.** Basic statistics of mass spectrum data results

## Sample repeatability test

For bioreplicated or technical replicated samples, it is necessary to check whether the quantitative results of the bioreplicated or technical replicated samples are statistically consistent. Three statistical analysis methods, principal component analysis (PCA), relative standard deviation (RSD) and Pearson's Correlation Coefficient, were used to evaluate the quantitative repeatability of proteins. Figure 2 shows the results of protein quantitative principal component analysis for all samples. In the figure, the better the degree of aggregation among repeated samples, the better the quantitative repeatability. FIG. 3 shows the box plot of the relative standard deviation (RSD) of quantitative protein values between repeated samples. The smaller the overall RSD value, the better the quantitative repeatability. Figure 4 is a heat map for calculating Pearson correlation coefficients between all sample pairings. This coefficient is a measure of the degree of linear correlation between two sets of data: the closer the Pearson coefficient is to -1, the closer it is to 1, the positive correlation, and the closer it is to 0, the correlation is not.


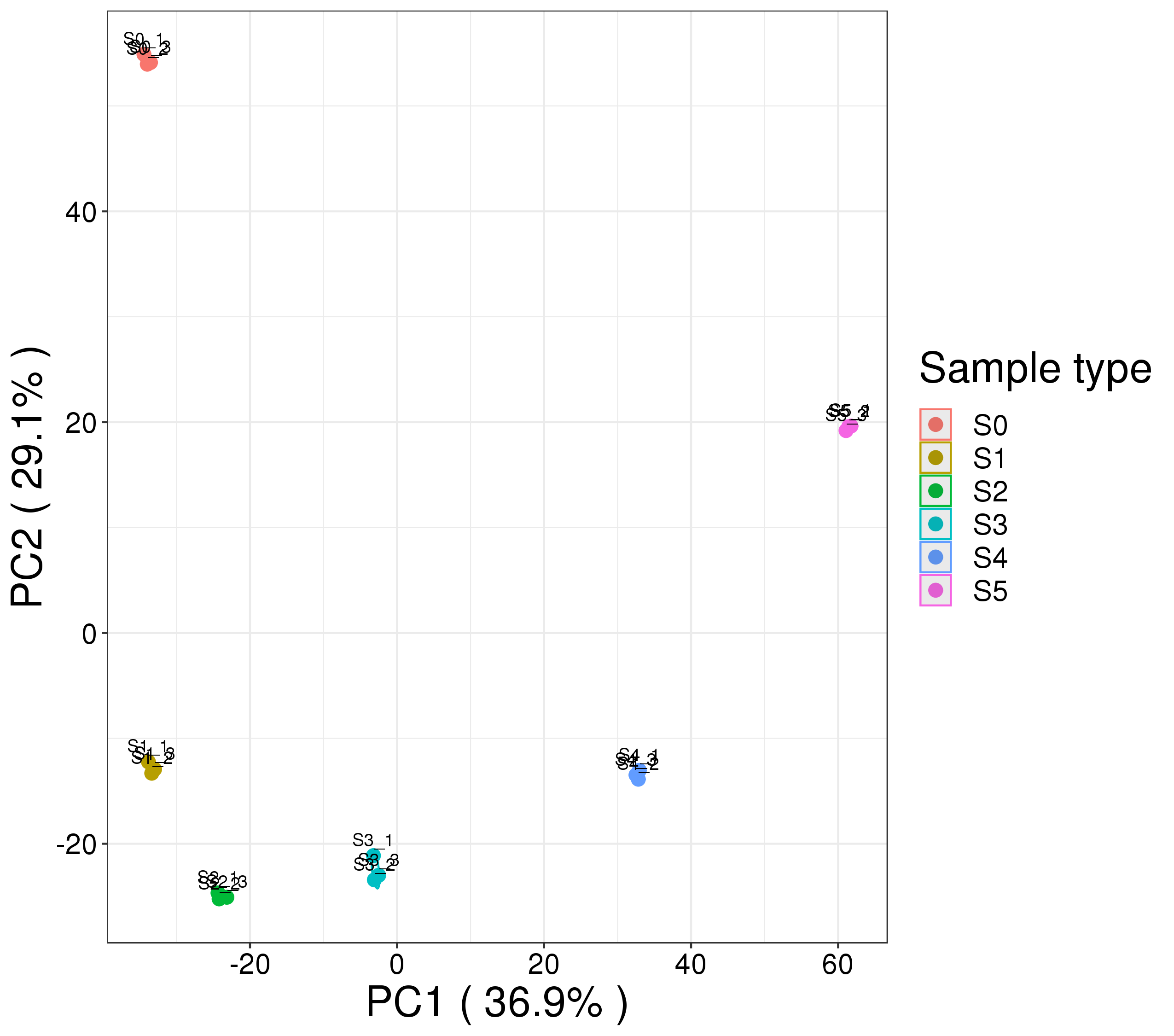


**Figure 2.** Two-dimensional scatter plot of principal component analysis of protein quantification between repeated samples


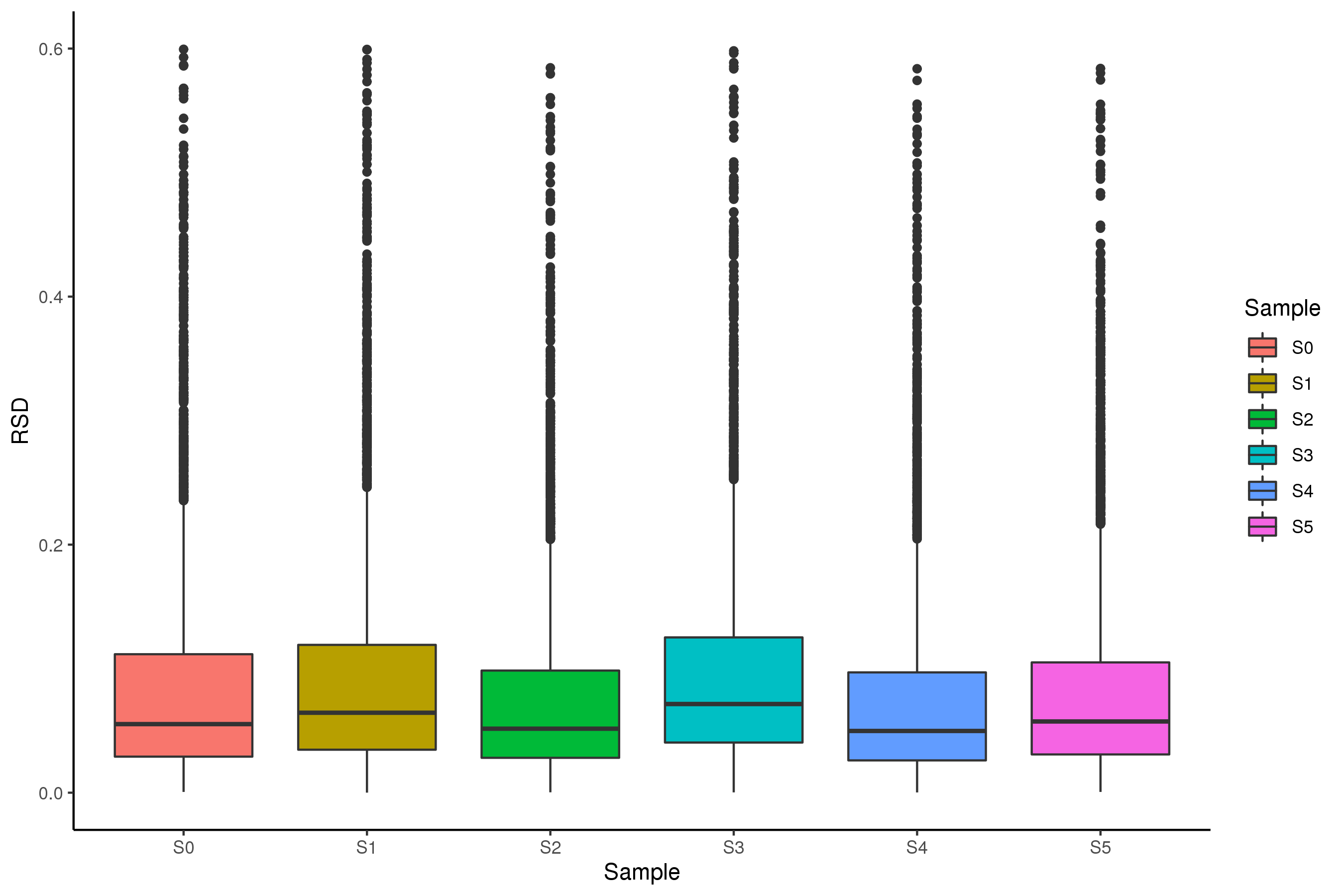


**Figure 3.** Box diagram of protein quantitative RSD distribution among repeated samples


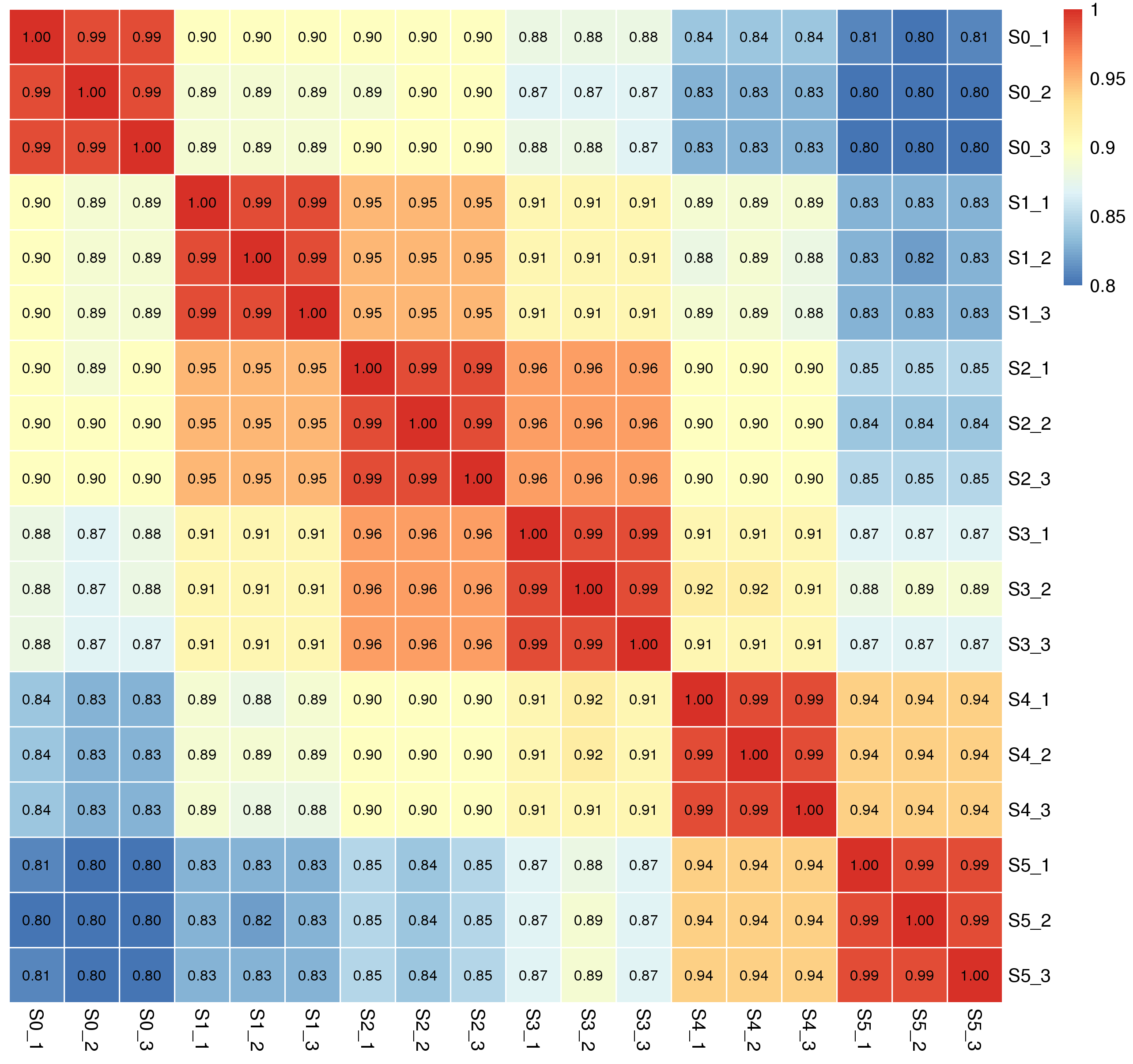


**Figure 4.** Pearson correlation coefficient heat map of protein quantification between pairwise samples

1. **Quality evaluation of metabolome data**

**Analysis of total ion flow diagram**

  In the process of instrument analysis, a quality control sample is inserted every 10 test analysis samples, and the stability of the instrument during the project test can be judged by overlapping display analysis of the total ion flow diagram (TIC) of the same quality control sample for essential spectrum detection and analysis. The high stability of the instrument provides an important guarantee for the repeatability and reliability of data.

[
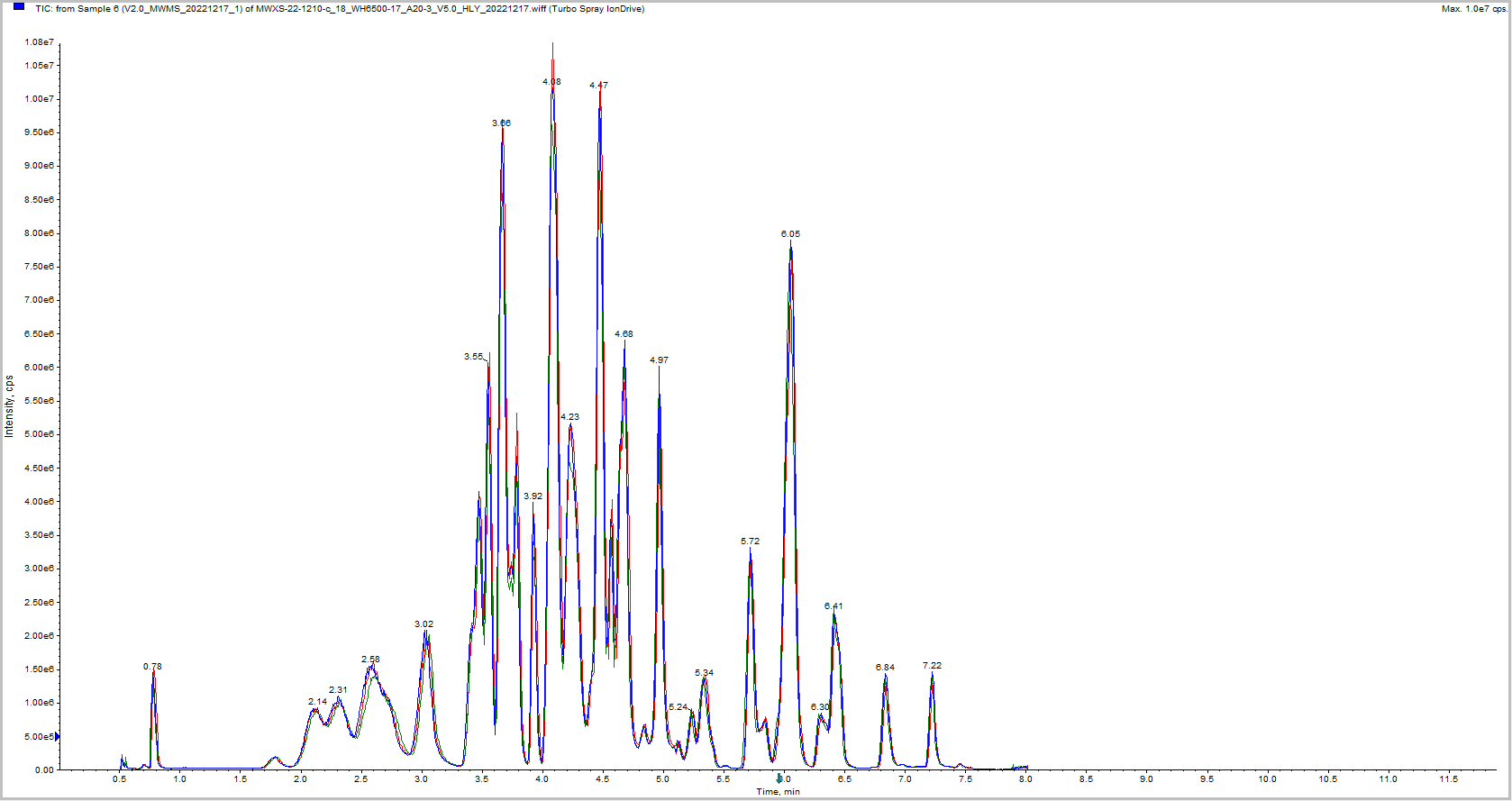
](file:///C:\Users\choccy\Desktop\%E5%A4%9A%E7%BB%84%E5%AD%A6\img\MWXS-22-1210-c_QC_MS_tic_overlap.png)

**TIC overlay chart**

Note: The curve overlap of the total ion flow detected by metabolites is high, that is, the retention time and peak intensity are consistent, indicating that the signal stability is better when the same sample is detected at different times by mass spectrometry. The high stability of the instrument provides an important guarantee for the repeatability and reliability of data.

**Distribution diagram of CV value of QC samples**

CV value, also known as Coefficient of Variation, is the ratio of the standard deviation of the original data to the average of the original data, which can reflect the degree of data dispersion. The Empirical Cumulative Distribution Function (ECDF) can be used to analyze the frequency of CV occurrence of substances that are smaller than the reference value. The higher the proportion of substances with lower CV value in QC samples, the more stable the experimental data: The proportion of QC samples with CV value less than 0.3 was higher than 80%, indicating that the experimental data was stable. The proportion of QC samples with CV value less than 0.2 is higher than 80%, indicating that the experimental data is very stable.

[
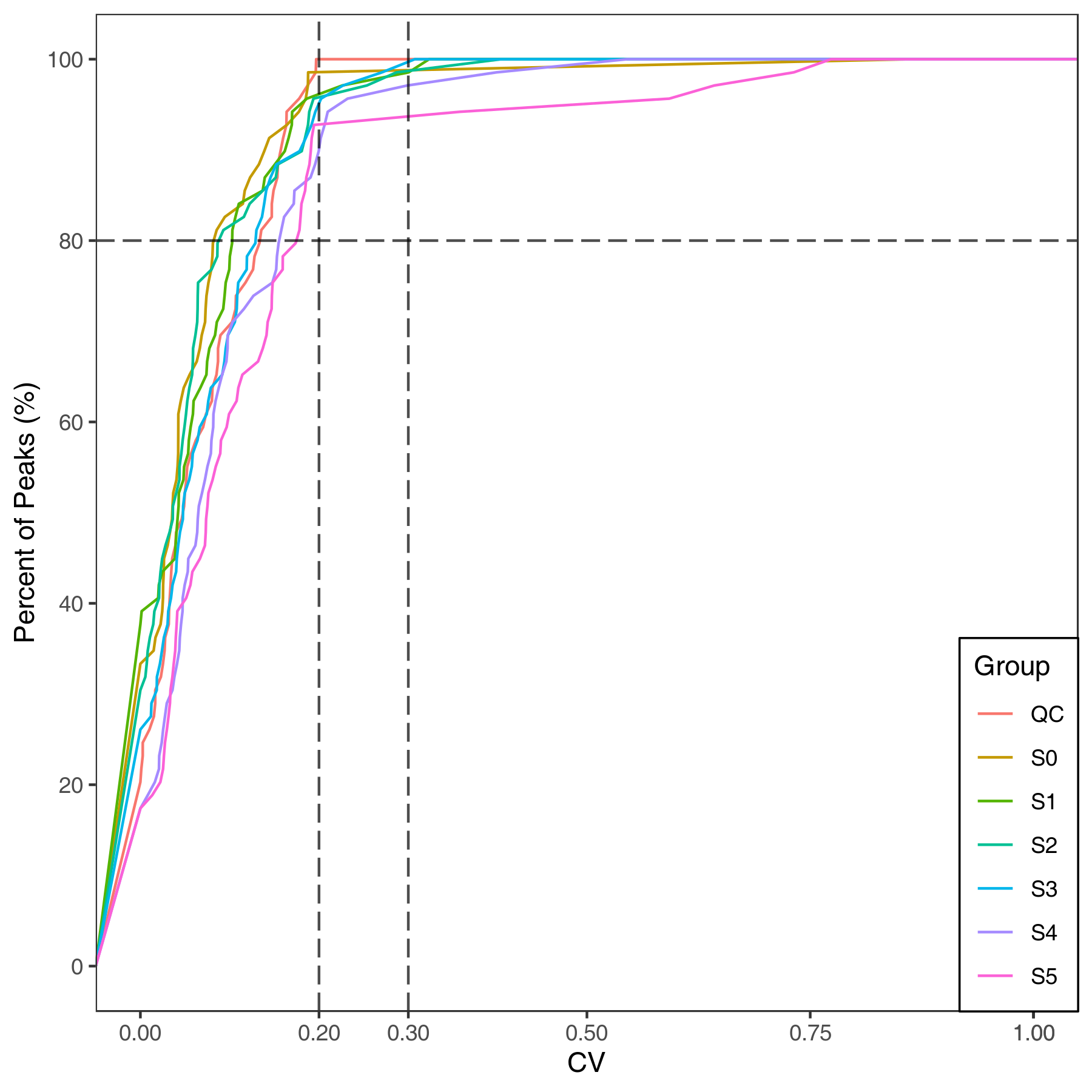
](file:///C:\Users\choccy\Desktop\%E5%A4%9A%E7%BB%84%E5%AD%A6\img\all_CV_ECDF.png)

**CV distribution in each group of samples**

Note: The horizontal coordinate represents the CV value, the vertical coordinate represents the proportion of the number of substances less than the corresponding CV value in the total number of substances, different colors represent different grouped samples, QC is the quality control sample, in which the CV value corresponding to the two reference lines perpendicular to the X-axis is 0.2 and 0.3, and the number of substances corresponding to the reference lines parallel to the X-axis accounts for 80% of the total number of substances.
