# Supplementary material for: Integrated proteomic, transcriptomic, and metabolomic profiling reveals that the gibberellin–abscisic acid hub runs flower development in the Chinese orchid Cymbidium sinense
Source: Hortic Res. 2024 Mar 12;11(5):uhae073. doi: 10.1093/hr/uhae073 (PMC11088716; doi:10.1093/hr/uhae073)
Supplement: Web_Material_uhae073 [file web_material_uhae073.zip › Supplementary figures.docx]

**Integrated proteomic, transcriptomic and metabolomic profiling reveals that GA-ABA hub runs the flower development in Chinese orchid *Cymbidium sinense***

Sagheer Ahmad^1,†^, Chuqiao Lu^1,†^, Jie Gao^1^, Yonglu Wei^1^, Qi Xie^1^, Jianpeng Jin^1^, Genfa Zhu^1,2^ and Fengxi Yang^1,2*^

^1^ Guangdong Key Laboratory of Ornamental Plant Germplasm Innovation and Utilization, Environmental Horticulture Research Institute, Guangdong Academy of Agricultural Sciences, Guangzhou, 510640, P.R. China

^2^ Guangdong Laboratory for Lingnan Modern Agriculture, Guangzhou, 510640, P.R. China


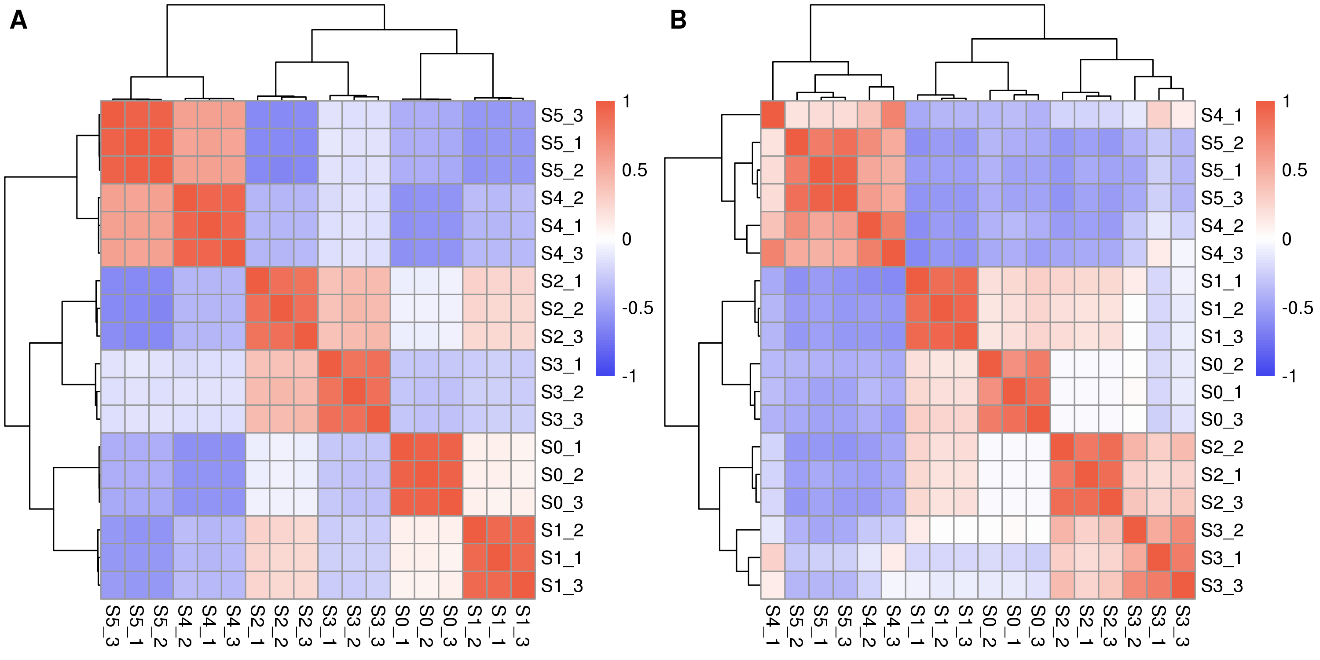


**Supplementary figure 1.** (A) Clustering heat map of quantitative pearson correlation coefficient of proteome between pairs of samples. (B) Clustering heat map of quantitative pearson correlation coefficient of transcriptome between pairs of samples.


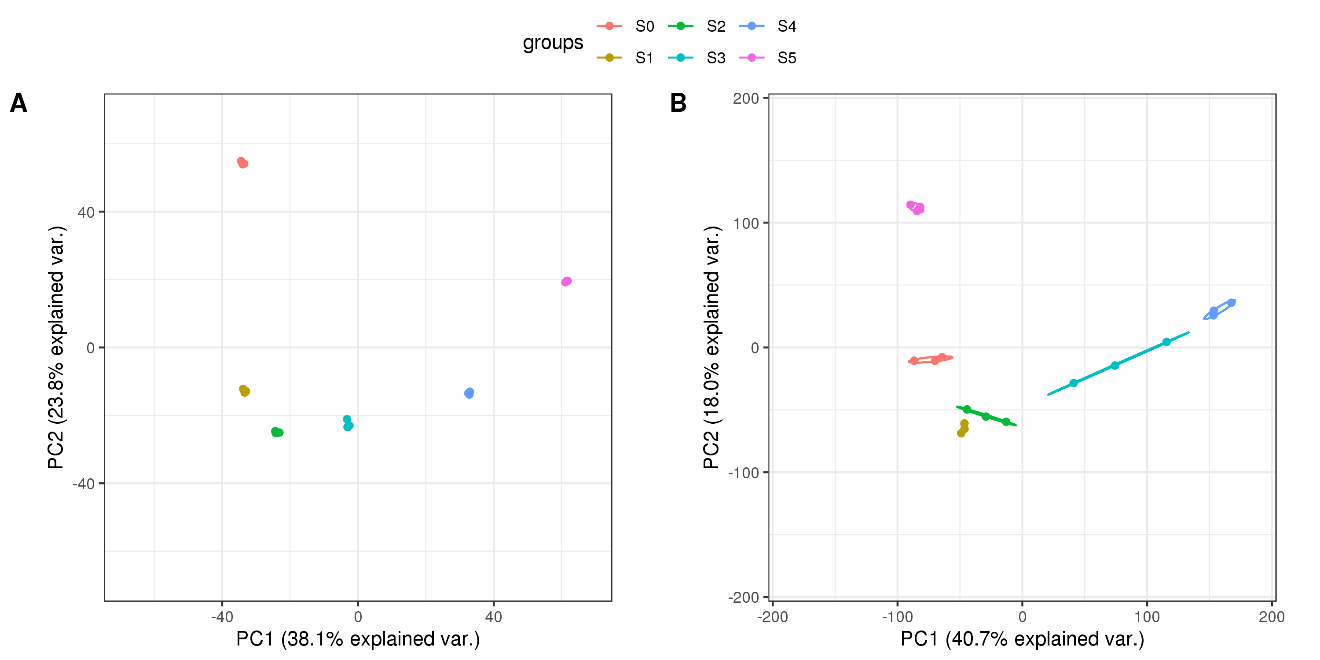


**Supplementary figure 2**. (A) Quantitative PCA scatter plot of all samples at the proteome level. (B) Quantitative PCA scatter plot of all samples at the transcriptome level.


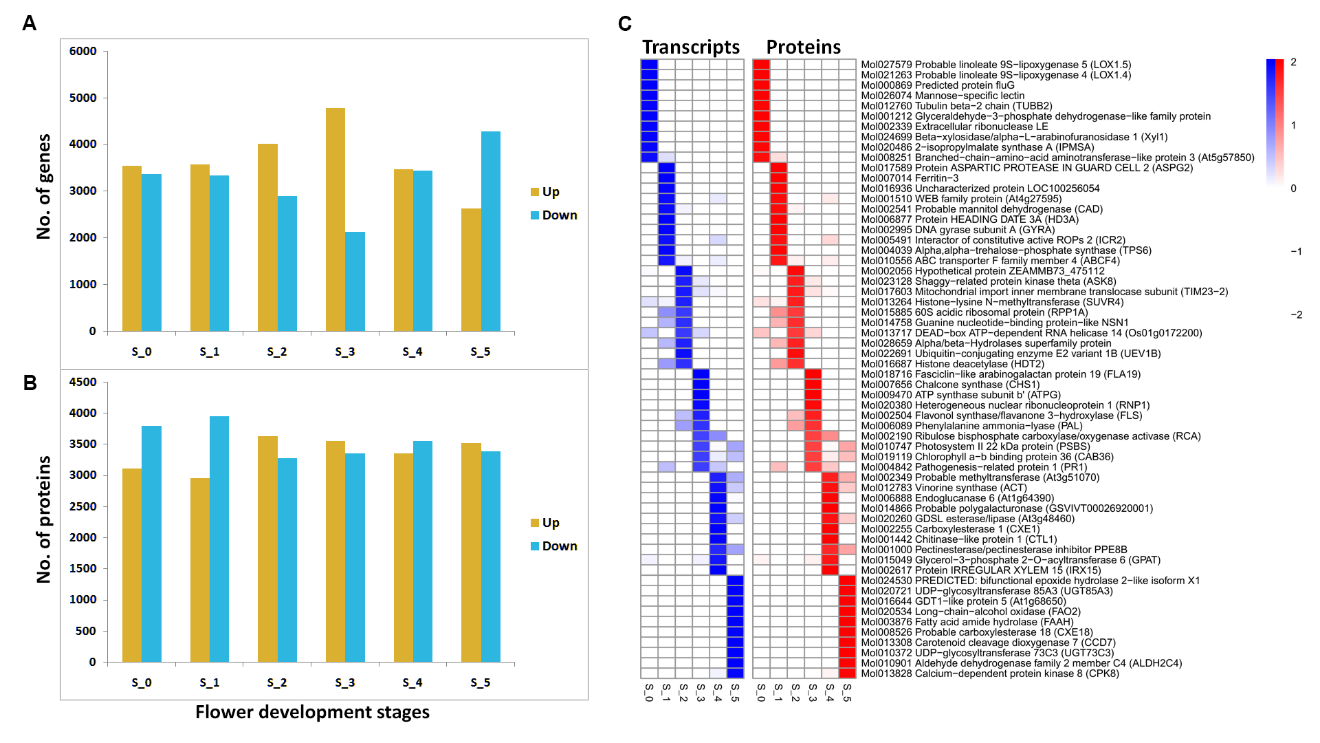


**Supplementary figure 3.** (A) Total number of upregulated and downregulated transcripts. (B) Total number of upregulated and downregulated proteins. (C) Top 10 highly significant transcripts with corresponding protein concentrations.


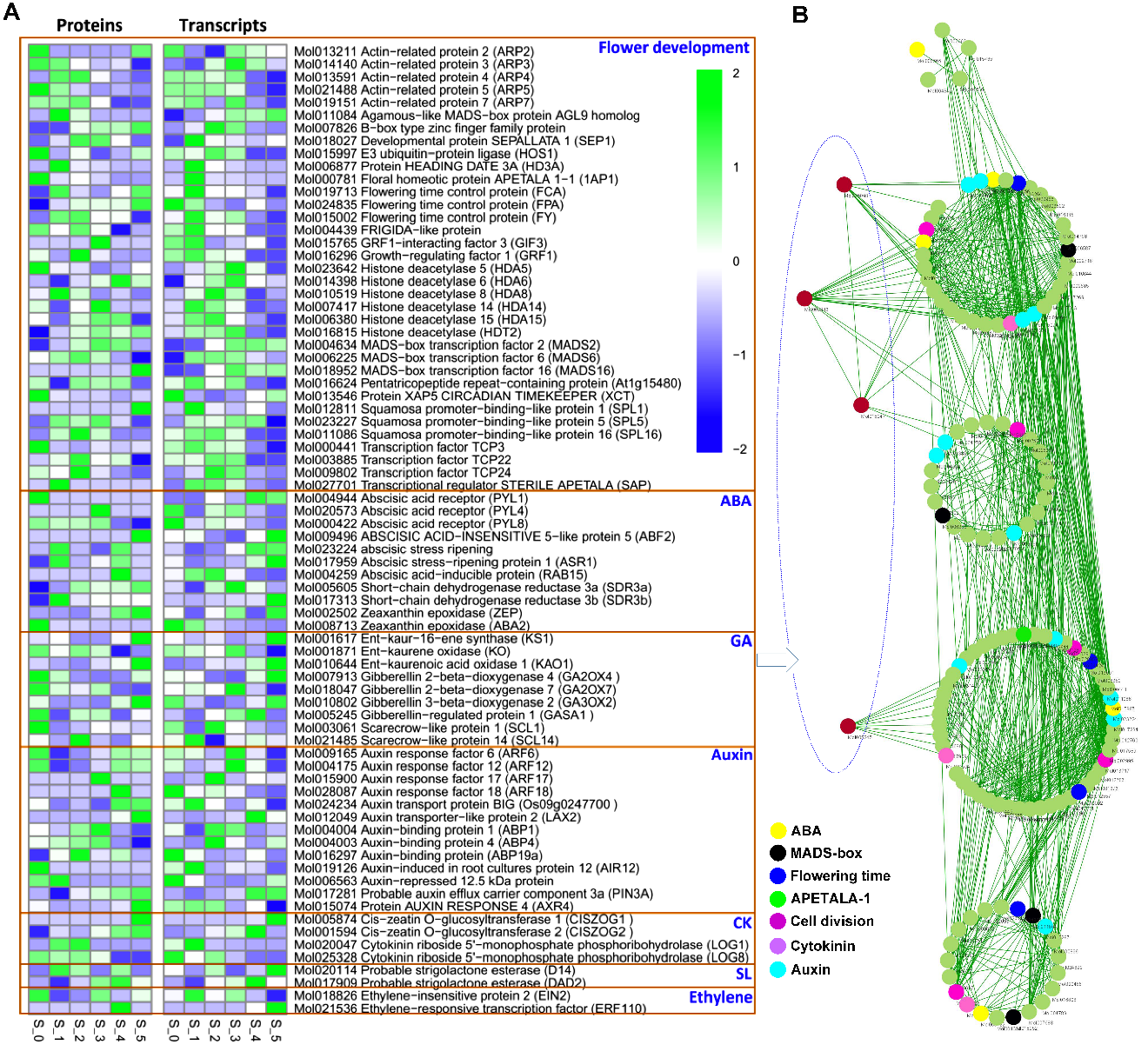


**Supplementary figure 4.** (A) Overview of the 78 potential transcripts with their corresponding protein concentrations. (B) The gene interaction network of these transcripts based on WGCNA analysis.


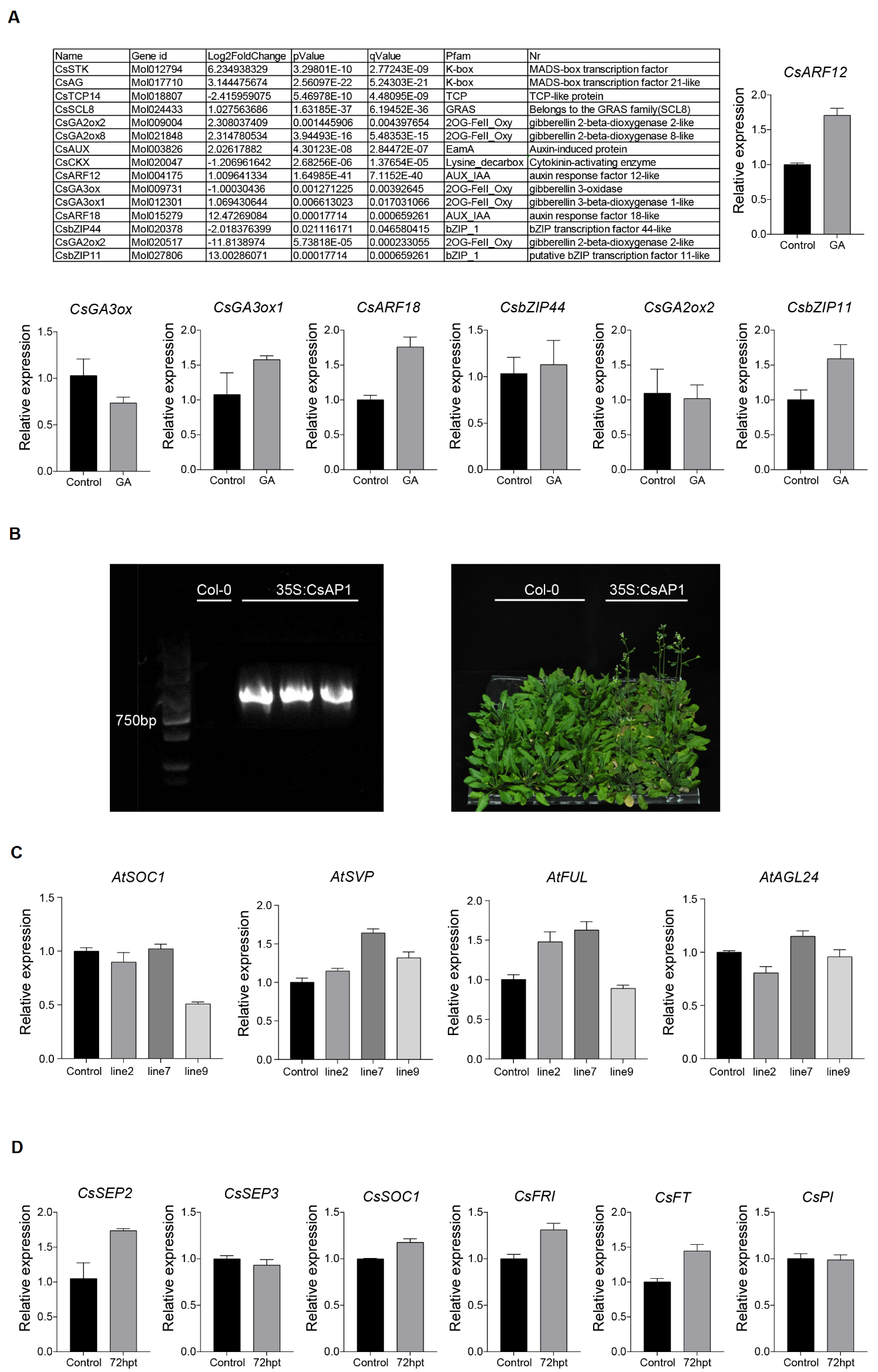


**Supplementary figure 5.** (A) List of genes with log2FC (fold change) expressions and annotations, and the bar graphs of their qRT-PCR expressions after exogenous GA application. (B) The overexpression of *CsAP1* in Arabidopsis. (C) The qRT-PCR expression of selected genes after overexpression of *CsAP1* in Arabidopsis. (D) The qRT-PCR expression of selected genes after transient overexpression of *CsAP1* in the protoplast of *C. sinense*.
